# Tumor cell-intrinsic MELK enhanced CCL2-dependent immunosuppression to exacerbate hepatocarcinogenesis and confer resistance of HCC to radiotherapy

**DOI:** 10.1186/s12943-024-02049-0

**Published:** 2024-07-05

**Authors:** Bufu Tang, Jinyu Zhu, Yueli Shi, Yajie Wang, Xiaojie Zhang, Biao Chen, Shiji Fang, Yang Yang, Liyun Zheng, Rongfang Qiu, Qiaoyou Weng, Min Xu, Zhongwei Zhao, Jianfei Tu, Minjiang Chen, Jiansong Ji

**Affiliations:** 1 Key Laboratory of Imaging Diagnosis and Minimally Invasive Intervention Research, School of Medicine, Lishui Hospital, Zhejiang University, Lishui, 323000 China; 2 Department of Radiology, Sir Run Run Shaw Hospital, Zhejiang University School of Medicine, Hangzhou, 310016 China; 3 Department of Radiation Oncology, Zhongshan Hospital Affiliated to Fudan University, Shanghai, China; 4 Key Laboratory of Carcinogenesis and Translational Research (Ministry of Education/Beijing), Department of Nuclear Medicine, Peking University Cancer Hospital & Institute, Peking University, Beijing, 100142 China; 5 Department of Respiratory and Critical Care Medicine, The Fourth Affiliated Hospital, School of Medicine, Zhejiang University, Yiwu, 322000 China; 6 Institute of Imaging Diagnosis and Minimally Invasive Intervention Research, The Fifth Affiliated Hospital of Wenzhou Medical University, Lishui, 323000 China; 7 Clinical College of The Affiliated Central Hospital, Lishui University, Lishui, 323000 China

**Keywords:** Hepatocellular carcinoma (HCC), miR-505, STAT3, CCL2, Tumor-associated macrophage (TAM), Radiotherapy (RT)

## Abstract

**Background:**

The outcome of hepatocellular carcinoma (HCC) is limited by its complex molecular characteristics and changeable tumor microenvironment (TME). Here we focused on elucidating the functional consequences of Maternal embryonic leucine zipper kinase (MELK) in the tumorigenesis, progression and metastasis of HCC, and exploring the effect of MELK on immune cell regulation in the TME, meanwhile clarifying the corresponding signaling networks.

**Methods:**

Bioinformatic analysis was used to validate the prognostic value of MELK for HCC. Murine xenograft assays and HCC lung metastasis mouse model confirmed the role of MELK in tumorigenesis and metastasis in HCC. Luciferase assays, RNA sequencing, immunopurification–mass spectrometry (IP-MS) and coimmunoprecipitation (CoIP) were applied to explore the upstream regulators, downstream essential molecules and corresponding mechanisms of MELK in HCC.

**Results:**

We confirmed MELK to be a reliable prognostic factor of HCC and identified MELK as an effective candidate in facilitating the tumorigenesis, progression, and metastasis of HCC; the effects of MELK depended on the targeted regulation of the upstream factor miR-505-3p and interaction with STAT3, which induced STAT3 phosphorylation and increased the expression of its target gene CCL2 in HCC. In addition, we confirmed that tumor cell-intrinsic MELK inhibition is beneficial in stimulating M1 macrophage polarization, hindering M2 macrophage polarization and inducing CD8 + T-cell recruitment, which are dependent on the alteration of CCL2 expression. Importantly, MELK inhibition amplified RT-related immune effects, thereby synergizing with RT to exert substantial antitumor effects. OTS167, an inhibitor of MELK, was also proven to effectively impair the growth and progression of HCC and exert a superior antitumor effect in combination with radiotherapy (RT).

**Conclusions:**

Altogether, our findings highlight the functional role of MELK as a promising target in molecular therapy and in the combination of RT therapy to improve antitumor effect for HCC.

**Supplementary Information:**

The online version contains supplementary material available at 10.1186/s12943-024-02049-0.

## Background

Hepatocellular carcinoma (HCC), the most prevalent form of primary liver cancer, ranked as the sixth most common malignant tumor in the world and the third leading cause of cancer-associated mortality worldwide in 2020 according to WHO statistics (https://gco.iarc.fr/today/fact-sheets-cancers), representing one of the most prominent global health problems today [1]. To prolong the life of HCC patients and improve their prognosis, a series of strategies, including hepatic resection, liver transplantation, transarterial chemoembolization (TACE), chemotherapy, radiotherapy (RT) and immunotherapy, have been widely tried and employed [2, 3]. Nevertheless, the carcinogenic process of HCC, as a highly heterogenetic disease, is affected by elusive molecular mechanisms and a complicated tumor microenvironment (TME), and the progression is rapid, which limits the effectiveness of these treatment options; thus, the prognosis of HCC patients is still not optimistic [4, 5]. Further investigation of the cellular mechanism of HCC development and the associated signaling pathways is urgently needed to unearth novel potential therapeutic targets for effective HCC treatment.

Maternal embryonic leucine zipper kinase (MELK) is a member of the AMP-related serine-threonine kinase family and participates in multiple cellular and biological processes, such as cell proliferation, cell cycle, stem cell self-renewal and apoptosis inhibition [6, 7]. In recent years, increasing evidence has confirmed that MELK serves as an oncogene in multiple cancer types, including colorectal cancer [8], breast cancer [9] and lung cancer [7]. Furthermore, MELK was also found to be essential for early HCC recurrence [10]. Nevertheless, the specific regulatory mechanism of MELK in the tumorigenesis and progression of HCC has not yet been fully clarified; this information is crucial for novel treatment strategy exploration.

The tumor microenvironment (TME) is a complex and continuously evolving entity containing multiple cell types, especially innate and adaptive immune cells, including macrophages, T cells and B cells, which have essential effects on both pro- and antitumorigenic effects in HCC [11]. Macrophages are a critical component of the TME and can be polarized into inflammatory M1 and immunosuppressive M2 phenotypes. High infiltration of TAMs in tumors is usually correlated with a poor prognosis of patients, and the TME mainly potentiates the polarization of specific TAM subsets to maintain tumor immunosuppression and treatment resistance, thereby supporting tumor initiation and progression [12–14]. Conversely, the presence of T cells, especially cytotoxic T cells (CD8+), plays a critical role in detecting abnormal tumor antigens expressed on tumor cells for targeted tumor cell destruction [11]. Notably, a recent study unveiled that MELK was correlated with various immune cells, including CD8 + T cells, CD4 + T cells, and macrophages, in HCC through bioinformatics analysis [15], and our previous research also confirmed that treatment with OTS167, an inhibitor of MELK, effectively suppressed the infiltration of macrophages and interfered with their polarization in colitis-propelled carcinogenesis [16]. However, the influence of MELK on immune cell infiltration, how MELK expression functions in TAMs polarization and T-cell recruitment in the TME to facilitate the tumorigenesis and progression of HCC, and the specific mechanism still need further exploration and clarification.

RT is actively used in clinical practice as a crucial therapeutic strategy for unresectable HCC [17]. The clinical efficacy of RT is attributed to its direct tumor cell killing effect caused by DNA damage [18] and indirect improvement in local and distant tumor control mediated by its enhancement of tumor-specific immunity [19]. However, some studies suggest that RT may also induce the cancer-promoting effect of the immunosuppressive tumor microenvironment, which is related to adverse outcomes in cancer patients [20]. Combining potential therapeutic targets which is helpful to effectively enhance the specific immune response activity against tumors to reverse the adverse immunosuppression caused by RT may be an effective breakthrough point to improve the curative effect of RT and improve the prognosis of HCC patients in clinical practice. Interestingly, evidence indicates that MELK also functions in regulating the radioresistance of glioma [21] and interfering with DNA damage tolerance in proliferating cancer cells [22]. Based on this, targeting MELK in HCC to enhance the antitumor effect of RT may be a promising way forward, but the role and mechanism of MELK in RT regulation of HCC needs further clarification.

In the present study, we focused on elucidating the functional consequences of MELK expression in the tumorigenesis, progression and metastasis of HCC, clarifying the corresponding molecular mechanism, identifying upstream regulators, and exploring downstream essential molecules and signaling networks. In addition, the effect of MELK on immune cell regulation in the TME and potential regulatory signaling were investigated to clarify its therapeutic value in combination with RT for HCC and provide new ideas for developing promising treatment strategies for HCC.

## Methods

### Data acquisition

In this study, mRNA-seq data of patients with HCC and corresponding clinical information were obtained from The Cancer Genome Atlas (TCGA) (https://www.cancer.gov/about-nci/organization/ccg/research/structural-genomics/tcga), ICGC (https://dcc.icgc.org/), and GEO (https://www.ncbi.nlm.nih.gov/geo/index.cgi) databases. The PITA (http://genie.weizmann.ac.il/pubs/mir07/mir07_dyn_data.html), miRanda (http://www.bioinformatics.com.cn/local_miranda_miRNA_target_prediction_120) and TargetScan (http://www.targetscan.org/) databases were incorporated into the potential targeted miRNA prediction analysis. The above databases are open to the public and are freely available, and this study strictly followed the access policy and publishing guidelines of the databases.

### Independent predictive factor identification and nomogram construction

Univariate Cox regression analyses were performed to identify the prognostic predictors of patients with HCC. Based on the confirmed independent factors, a nomogram was established using “rms” R software. Corresponding calibration curves were generated to assess the consistency between the predicted results of the nomogram and the actual results. And we further established a nomogram program (https://zjuprediction.shinyapps.io/DynNomapp/) for HCC survival prediction by entering the data of independent prognostic factors online.

### Functional enrichment and gene set enrichment analyses (GSEA)

Differentially expressed genes (DEGs) in HCC-LM3 cells with or without MELK knockdown, identified using the limma R package with |log2(fold change) |>1 and adjusted *P* value < 0.05, were included in Kyoto Encyclopedia of Genes and Genomes (KEGG) analysis and Gene Ontology (GO) analysis to explore the potential enriched biological processes and signaling pathways. The potential biological processes closely related to MELK expression from the TCGA-LIHC cohort were further investigated using GSEA. The adjusted *P* value is calculated by the false discovery rate (FDR) correction based on the Benjaminiand Hochberg (BH) method and the formula is listed as follows: Adjusted *P* value = P *(m/k), in which m represents the times of tests and k represents the rank corresponding to the *p* value of one of the test results.

### HCC tissue acquisition

Sixty-eight HCC tissues and paired tumor-adjacent normal tissues were obtained from Lishui Hospital of Zhejiang University and confirmed by pathological diagnosis. Written informed consent was acquired from all patients prior to the study. This study was approved by the Research Ethics Committee of the Lishui Hospital of Zhejiang University and adhered to the Declaration of Helsinki principles.

### Cell culture and lentiviral infection

The cell lines used in this study, including human HCC cell lines (SK-HEP1, HCC-LM3, MHCC-97 H and Hepa1-6) and human embryonic kidney cells (239T), were all acquired from the American Type Culture Collection (Manassas, VA, USA). The cells were maintained in Dulbecco’s Modified Eagle Medium (DMEM) supplemented with 10% (v/v) fetal bovine serum (FBS) and 100 U/mL penicillin/streptomycin and cultured in a constant temperature sterile incubator at 37 ℃ with an atmosphere of 5% CO2. Recombinant lentiviruses expressing scramble-shRNA (shSCR) and MELK-shRNA (shMELK) were generated by GenePharma (Shanghai, China). Cells were seeded in a 6-well plate at a density of 5 × 10^5^ cells/well and infected with the concentrated viruses (8 µg/mL). Then, infected cells were sorted for target expression, and HCC cells stably transfected with shSCR or shMELK were cultured for subsequent experiments. shRNA sequences are listed in the supporting information.

### Design and transfection of sgMELK

The sgRNA against MELK by CRISPOR web based tool with minimal off target activity was designed and synthesized by Tsingke (Tsingke, Beijing, CN), cloned in non-viral pSpCas9(BB)-2 A-Puro (PX459) V2.0 (sgMELK) and confirmed by the molecular digestion. And non-targeting control gRNA CRISPR vector was used for control.Target sequences are available in the supporting information. HCC cells were transfected with sgMELK or control vector. Lipofectamine 3000 (ThermoFisher Scientific, MA, USA) was used according to manufacturer’s instructions for cell transfection. Forty-eight hours after transfection, replacing the culture solution with fresh culture solution containing Puromycin (2 µg/mL) and continuing to culture for another 48 h. Then the cells were seen under an inverted microscope and the still alived ones were successfully transfected. Dilution culture was carried out on the infected and screened cells with mixed clones, and cell clones grown from single cells were selected to culture for subsequent experiments.

### Truncation body construction

The full nucleotide sequence of STAT3 and MELK from plasmids (pXJ40-myc-STAT3 and pXJ40-myc-MELK) were used as the template for PCR to amplify the truncated mutants using Q5^®^ High-Fidelity 2× Master Mix (New England Biolabs Inc., USA). For all the truncated mutants, sequence of the “TGA” was added in the ending of the sequence as the termination codon to terminate translation. The PCR products of the truncated STAT3 mutants were digested using FastDigest HindIII and XmaI restriction enzymes (Thermo Scientific, USA), and MELK mutants were digested using BamHI and BglII restriction enzymes (Thermo Scientific, USA). The vector was also digested using the corresponding restriction enzymes. Then, according to the instructions, a DNA linking kit was used to ligate the digested PCR product and vector. The resultant plasmids were transformed into DH5α Escherichia coli, and then sent for sequencing to verify that the constructs were successful.

### Cell proliferation assay

Cell Counting Kit-8 (CCK-8) assays and EdU (5-ethynyl-2’-deoxyuridine) assays were carried out to evaluate cell proliferation. The CCK-8 assay was performed as follows: HCC cells stably transfected with shSCR or shMELK (including SK-HEP1 and HCC-LM3) were seeded into a 96-well tissue culture plate in complete medium (approximately 1 × 10^3^ cells/well), and 10 µL of CCK-8 reagent was added to each well 2 h before detecting the OD at 450 nm at the indicated times using a microplate reader (Bio-Rad, Berkeley, CA, USA). Each group included three replicate wells. For the EdU assay, briefly, HCC cells stably transfected with shSCR or shMELK were cultured in 6-well plates at a density of 5 × 10^5^ cells/well and incubated overnight. Then, 10 µM EdU solution was added to the cells 2 h before detection. The proliferation of cells was evaluated using the Cell-Light EdU Cell Proliferation Detection kit (RiboBio, Guangzhou, China) according to the manufacturer’s protocol and visualized under a fluorescence microscope (Nikon, Japan).

### Colony formation assay

HCC cells stably transfected with shSCR or shMELK were placed in a 6-well plate at a density of 500 cells/well and cultured in a constant temperature incubator for 2 weeks. Then, the cells were fixed with 4% paraformaldehyde and stained with 0.1% crystal violet, and the relative number of colonies was counted under a microscope.

### Transwell migration assay

HCC cells stably transfected with shSCR or shMELK were treated with mitomycin C (10 µg/mL) for 1 h prior to the transwell assay to exclude the influence of proliferation on migration. Then, the cells were suspended in serum-free DMEM at a density of 6 × 10^5^ cells/ml. A 200 µL cell suspension was added to the upper chamber of a 24-well culture plate equipped with an 8.0 μm pore size polycarbonate membrane, and 700 µL DMEM containing 10% FBS was added to the lower chamber. After incubation of cells at 37℃ for 18 h, the cells on the upper side of the membrane were removed using cotton swabs, and the cells passing through the membrane were stained with 0.4% trypan blue and counted under an optical microscope.

### Immunofluorescence (IF) staining

HCC cells stably transfected with shSCR or shMELK were grown on glass slides in a 6-well plate. After being treated with 4% paraformaldehyde, 0.3% Triton X-100 and goat serum in turn, the cells were incubated with the corresponding primary antibodies at 4℃ overnight. Then, the cells were incubated with the corresponding Alexa Fluor-labeled secondary antibodies (Beyotime, Shanghai, China) at room temperature for 60 min in the dark and counterstained with 4’,6-diamidino-2-phenylindole (DAPI) for another 10 min. The fluorescence images were visualized and analyzed using a confocal laser scanning microscope (Leica Microsystems Inc., GER).

### Murine xenograft assay

To investigate the effect of MELK knockdown on HCC occurrence and development, athymic male BALB/c nude mice aged 5–6 weeks were randomly divided into the shSCR group and shMELK group (5 mice/group). HCC-LM3 cells stably transfected with shSCR or shMELK were suspended in PBS at a density of 1.5 × 10^8^ cells/mL, and 200 µL of cell suspension was subcutaneously injected into a side of the lower abdomen of each mouse. The groups were fed with adequate food and water under a 12-hour dark/12-hour light cycle and specific pathogen-free conditions. When subcutaneous tumors were palpable (nearly 3 mm), the volume of each tumor was measured every 3 days and calculated using the following formula: volume = 0.5 × length × width^2^. Three weeks after injection, the mice were euthanized by cervical dislocation under isoflurane anesthesia, and the xenograft tumors were removed for subsequent analysis.

To analyze the clinical effect of MELK knockdown in combination with RT on HCC tumorigenesis, male C57BL/6 mice aged 6–8 weeks (Shanghai SLAC Laboratory Animal Co., Ltd) were randomly assigned into several groups with different treatments (vector, shMELK, RT, shMELK + RT) (5 mice/group) and subcutaneously injected with 200 µl of Hepa1-6 cell suspension containing approximately 5 × 10^6^ cells transfected with shSCR or shMELK. When the subcutaneous tumors were palpable (nearly 3 mm), the mice in the RT and shMELK + RT groups received RT at 8 Gy every two days for a total of three times, and the volume of each tumor was measured every 3 days. Three weeks after injection, the mice were euthanized by cervical dislocation under isoflurane anesthesia, and the tumors were removed for subsequent experiments.

### Establishment of mouse orthotopic implantation models of HCC

Athymic male BALB/c nude mice aged 5–6 weeks were randomly divided into the shSCR group and shMELK group (5 mice/group). HCC-LM3 cells stably transfected with shSCR or shMELK were suspended in PBS containing Matrigel matrix (Corning, NY, USA) and injected into the livers of mice with a 100 µL cell suspension containing approximately 3 × 10^7^ cells [23, 24]. Three weeks after the injection, the mice were euthanized by cervical dislocation under isoflurane anesthesia, and the livers were removed for subsequent experiments.

### HCC lung metastasis mouse model construction

Athymic male BALB/c nude mice aged 5–6 weeks were randomly divided into the shSCR group and shMELK group (5 mice/group) and were injected with 200 µL of cell suspension containing approximately 1 × 10^6^ HCC-LM3 cells transfected with shSCR or shMELK via the tail vein. The progression of tumor lung metastasis in each mouse was detected weekly using an IVIS Spectrum in vivo imaging system (PerkinElmer, MA, USA).

### Immunohistochemical (IHC) staining and analysis

HCC-LM3 tumors were fixed in 4% paraformaldehyde for 24 h before being embedded in paraffin, and then the tumor tissues were cut into Sect. (4 × 4 μm). After deparaffinization and rehydration, the sections were incubated with methanol supplemented with 30% H2O2 to eliminate endogenous peroxidase activity, followed by antigen retrieval via heat induction. Then, the sections were blocked in PBS supplemented with 10% FBS for 45 min and incubated with the corresponding primary antibody at 4℃ overnight, followed by incubation with secondary biotinylated antibodies at room temperature for an additional 2 h. The sections were visualized using the OptiView DAB IHC Detection Kit (Thermo Scientific™, MA, USA) according to the manufacturer’s protocol and imaged under a light microscope.

The IHC score was calculated and determined using ImageJ software combining the intensity of specific staining with the proportion of labeled cells as described previously [25, 26]. That is, the specific staining intensity was defined as follows: 0 (negative), 1 (weakly positive), 2 (positive), and 3 (strongly positive). The IHC score was calculated using the following formula: IHC score = (% of strongly positive cells × 3) + (% of positive cells × 2) + (% of weakly positive cells × 1).

### Extraction and culture of bone marrow-derived macrophages (BMDMs)

Femurs and tibias of healthy C57BL/6 wild-type mice (aged 6–8 weeks) were flushed with PBS, and the suspension was passed through a 100 µL nylon cell filter (Falcon, USA). Then, density gradient centrifugation was performed using Lymphoprep (Axis Shield PoC AS, Oslo, Norway) to harvest bone marrow-derived monocytes. The cells were cultured in RPMI 1640 medium containing 10% (v/v) heat-inactivated FBS, 2 mM GlutaMAX, 100 U/mL penicillin/streptomycin and 20 g/mL M-CSF and incubated in a constant temperature sterile incubator at 37℃ for 7 days to obtain M-CSF-differentiated macrophages for subsequent experiments.

### Coculture system establishment

To mimic the generation of TAMs, BMDMs were cocultured with Hepa1-6 cells transfected with shSCR or shMELK in a 6-well transwell cocultivation system (0.4 μm pore size, Corning, USA), in which Hepa1-6 cells were cultured in the upper chamber and BMDMs were grown in the lower chamber. After 48 h of incubation, the cocultured macrophages were harvested to obtain TAMs for subsequent analyses.

### Flow cytometry (FCM) analysis

BMDMs and RAW264.7 cells cocultured with Hepa1-6 cells were harvested and made into single-cell suspensions, followed by staining with surface antibodies in FACS buffer for 30 min at 4℃ in the dark. Then, the stained cells were washed twice with PBS and resuspended in 200 µL of flow buffer. A FACSCalibur TM Flow Cytometry System was used for sample analysis, and FlowJoTM software (version 10.6.2) was used for FACS data analysis. Information on the antibodies used for staining is listed in the supporting information.

### Dual-luciferase reporter assay

The DNA sequence containing the predicted binding site with miR-505-3p in the 3’-untranslated region (3’ UTR)-MELK wild type (WT) or the 3’ UTR-MELK mutant (MT) was cloned into the pmirGLO dual-luciferase reporter vectors. Mutations in the miR-505-3p binding site were generated from 5’-TGTTGAC-3’ (MELK-WT) to 5’-GTGGTCA-3’ (MELK-MT). HCC cells were seeded in a 24-well plate at a density of 1 × 10^5^ cells/well and incubated for 24 h. Then, Lipofectamine 3000 (Invitrogen, USA) was used to cotransfect the recombinant plasmid pmirGLO-3’UTR-MELK WT or pmirGLO-3’UTR-MELK MT with miR-505-3p mimics or mimic NC into the cells. After incubation for 48 h, the luciferase activity was detected using a dual-luciferase reporter assay system (Vazyme, Nanjing, China) according to the manufacturer’s instructions for the Dual-Luciferase Reporter Gene Assay kit (Promega, WI, USA).

### Western blotting (WB)

Protein was extracted from HCC cell lines or tumors using RIPA Lysis and Extraction Buffer (Thermo Scientific™, MA, USA) containing 1% protease inhibitor cocktail (Cell Signaling Technology, MA, USA) and then separated into multiple bands by sodium dodecyl sulfate‒polyacrylamide gel electrophoresis (SDS‒PAGE, 10%) according to the molecular weight, followed by electrotransfer to a polyvinylidene fluoride (PVDF) membrane. Then, the membrane was blocked in 5% nonfat powdered milk at room temperature for 90 min and subsequently incubated with the corresponding primary antibody at 4℃ overnight, followed by incubation with the horseradish peroxidase (HRP)-conjugated secondary antibody at room temperature for another 2 h. The bands on the membrane were visualized using the iBright™ FL1500 Imaging System (Thermo Scientific™, MA, USA). The antibodies used in the study can be found in the supporting information.

### Quantitative real-time polymerase chain reaction (qRT‒PCR)

Total RNA was extracted from the treated cells using TRIzol^®^ reagent (Invitrogen, CA, USA) following the manufacturer’s protocol, followed by reverse transcription to synthesize cDNA using the RevertAid First Strand cDNA Synthesis kit (Thermo Scientific™, USA). Then, qRT‒PCR was carried out according to the manufacturer’s instructions for SYBR Green PCR Master Mix (Thermo Scientific™) using a LightCycler Roche480 (Roche). The mRNA expression for each target gene was normalized to that of the endogenous control (β-actin) via the 2^−ΔΔCt^ method. The test was repeated in triplicate. Sequencing of the primers used for the test is listed in the supporting information.

### Immunopurification–mass spectrometry (IP-MS)

293T cells expressing FLAG-MELK were lysed in IP buffer (20 mM Tris-HCl, pH 8.0, 150 mM NaCl, 2 mM EDTA and 1% Nonidet P-40) containing a protease inhibitor cocktail (Roche, Swiss). The lysates were then applied to an equilibrated FLAG column and incubated at 4 °C for 4 h, followed by washing with IP buffer and elution with FLAG peptides (Sigma–Aldrich). Fractions of the bed volume were collected and resolved through SDS–PAGE and silver staining. LC–MS/MS sequencing was then carried out for the gel bands by Qinglian Bio (Beijing, China).

### Coimmunoprecipitation (CoIP)

IP buffer supplemented with protease inhibitor cocktail and phosphatase inhibitor cocktail was used to extract proteins from cells. 30 µL of Pierce™ Protein A/G Magnetic Beads (#88,802, Thermo Fisher, USA) and 2 µL of MELK (#2274, Cell Signaling Technology), 2 µL of STAT3 (#12,640, Cell Signaling Technology), 0.5 µL of DYKDDDDK Tag (#14,793, Cell Signaling Technology) or 0.5 µL of normal rabbit IgG (#sc2027, Santa Cruz Biotechnology) were stirred for 10 min at room temperature, and cell lysates were then incubated with the antibody-conjugated magnetic beads at 4℃ overnight. Then, the magnetic beads were washed with PBST, and the conjugated protein was finally dissolved in 50 µL of IP buffer and 10 µL of 6× DNA loading buffer (Beyotime, Shanghai, China) and boiled at 100℃ for 8–10 min for immunoblotting analysis.

### Cytokine arrays

Serum from mice bearing Hepa1-6 tumors expressing shSCR or shMELK was collected via centrifugation of peripheral blood (3000 rpm, 10 min, 4℃). The expression of cytokines related to immune cell recruitment in the serum was assessed with a commercial protein array kit (Proteome Profiler Mouse Cytokine Array Kit, R&D system, ARY006) according to the manufacturer’s instructions. The expression of each tested target was calculated with ImageJ software (National Institutes of Health).

### Enzyme-linked immunosorbent assay (ELISA)

To detect the expression of CCL2 and IFN-γ in the serum of mouse models bearing Hepa1-6 tumors expressing shSCR or shMELK, ELISA was carried out using the Mouse CCL2/JE/MCP-1 ELISA Kit and Mouse IFN-γ ELISA Kit (Beyotime Biotechnology, Shanghai, China) according to the manufacturer’s instructions.

### Statistical analysis

Each experiment in this study was conducted at least in triplicate independently, and the results were analyzed using GraphPad Prism (version 8.3.0) and graphically displayed through R software (version 4.0.5). Student’s t test, Wilcoxon test and one-way ANOVA were adopted to evaluate the difference between groups. Data are presented as the mean value ± standard error of the mean (SEM). *P* < 0.05 was considered to imply statistical significance.

## Results

### MELK is elevated in HCC and correlates with a poor prognosis in patients with HCC

**Fig. 1:**
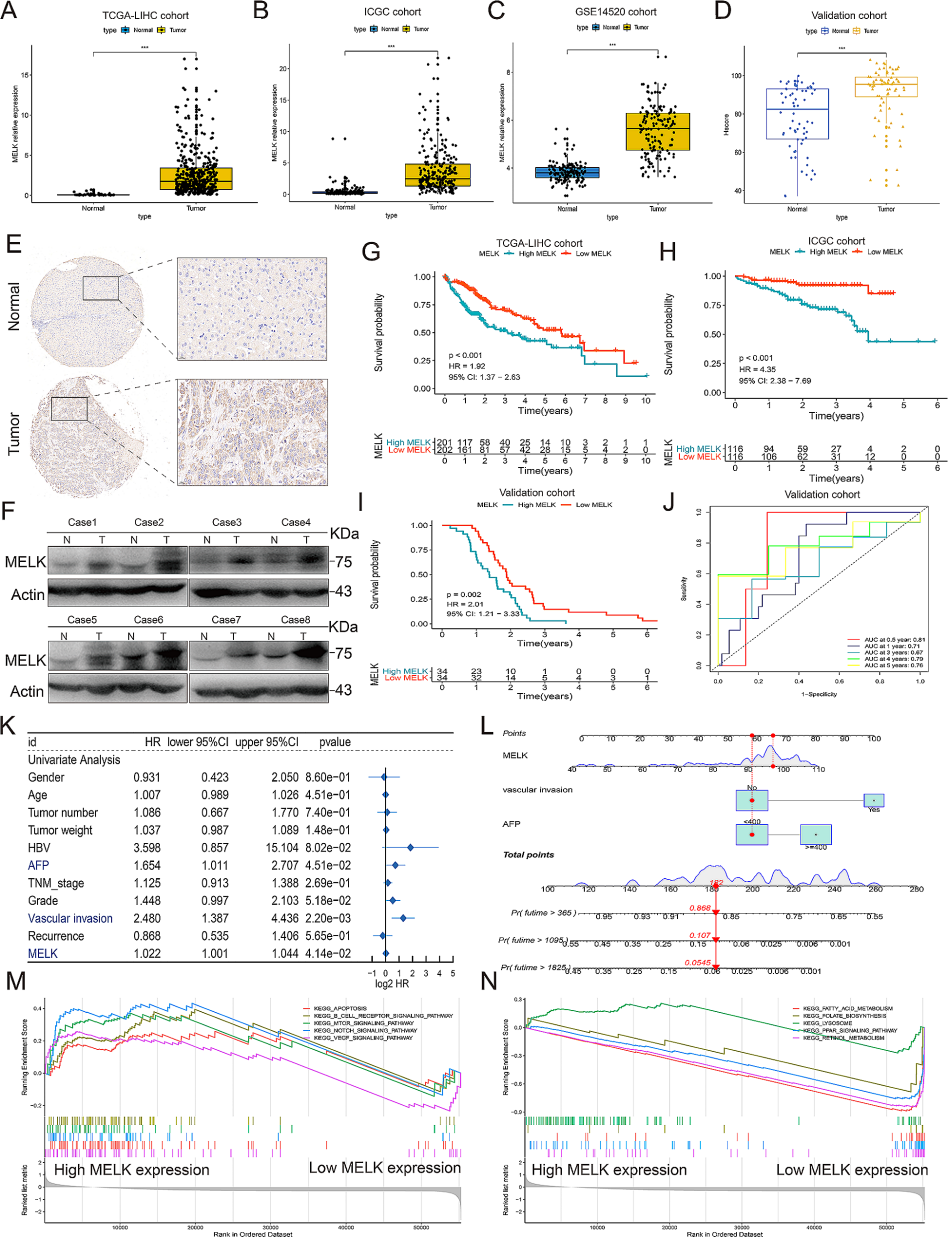
Upregulated MELK predicts a poor prognosis in HCC patients. (**A-C**) The expression difference between tumor tissues and corresponding normal tissues in the TCGA-LIHC cohort (A), ICGC cohort (B) and GSE14520 cohort (C). **(D)** An external validation cohort confirming the expression changes of MELK between HCC tissues and normal samples. **(E-F)** IHC staining (E) and Western blot (WB) assay (F) reflecting the expression characteristics of MELK in HCC tissues and normal samples. **(G-I)** Survival analysis showing the prognosis of HCC patients with high or low MELK expression in the TCGA-LIHC cohort (G), ICGC cohort (H) and validation cohort (I). **(J)** ROC curves confirming the prognostic predictive reliability of MELK expression in HCC patients in the validation cohort. **(K)** Identification of prognostic predictive factors of HCC. **(L)** The construction of a nomogram integrating the prognostic factors of HCC. **(M)** The signaling pathways are positively related to the high expression of MELK. **(N)** The signaling pathways negatively related to the high expression of MELK. *** *p* < 0.001, **** *p* < 0.0001

To investigate the role of MELK in regulating HCC tumorigenesis, we first sought to characterize the expression profiles of MELK in HCC. To this end, we conducted a holistic view of the expression characteristics of MELK in tumors in different organs from the TCGA database (Figure S1A-B), and analysis of the TCGA-LIHC cohort (Fig. 1A), ICGC cohort (Fig. 1B) and GSE14520 cohort (Fig. 1C) revealed that the expression of MELK in tumor tissues was significantly increased compared to that in normal tissues. We also collected 68 HCC samples as an external validation cohort, and the expression changes in MELK were in accordance with the above findings (Fig. 1D-F). In addition, HCC patients with high MELK expression had a worse prognosis than those with low MELK expression in both the TCGA-LIHC cohort (Fig. 1G and S1C) and the ICGC cohort (Fig. 1H), and a consistent result was shown in the validation cohort (Fig. 1I). The results of ROC analyses implied that the prognostic predictive performance of MELK expression was reliable (Figure S1D-E and 1 J).

Realizing the good predictive value of MELK on the prognosis of HCC patients, we further investigated the association between the MELK expression with traditional clinical prognostic features and observed poorer clinicopathological characteristics HCC patients with higher expression of MELK (Table S1). In addition, HBV infection, AFP, vascular invasion and MELK were confirmed to be the prognostic factors of HCC (Fig. 1K), followed by the construction of a nomogram based on these prognostic factors (Fig. 1L). We also identified the prognostic factors of HCC in the TCGA-LIHC cohort and constructed a nomogram (Figure S1F-G). The calibration curves revealed the good prognostic predictive value of the nomogram (Figure S1H-J). To further investigate the mechanism by which MELK affects the tumorigenesis and prognosis of HCC, we performed GSEA and found a positive correlation between MELK and well-known oncogenic pathways, including apoptosis, the mTOR signaling pathway and Notch signaling pathways [27, 28](Fig. 1M), and a negative correlation between MELK and fatty acid metabolism and the PPAR signaling pathway [29], which are important in maintaining the normal function of cells (Fig. 1N). Together, these findings support that MELK may serve as a driver of HCC tumorigenesis and is closely related to the poor prognosis of HCC.

### MELK inhibition impairs the tumorigenesis, progression and spontaneous lung metastasis of HCC

**Fig. 2:**
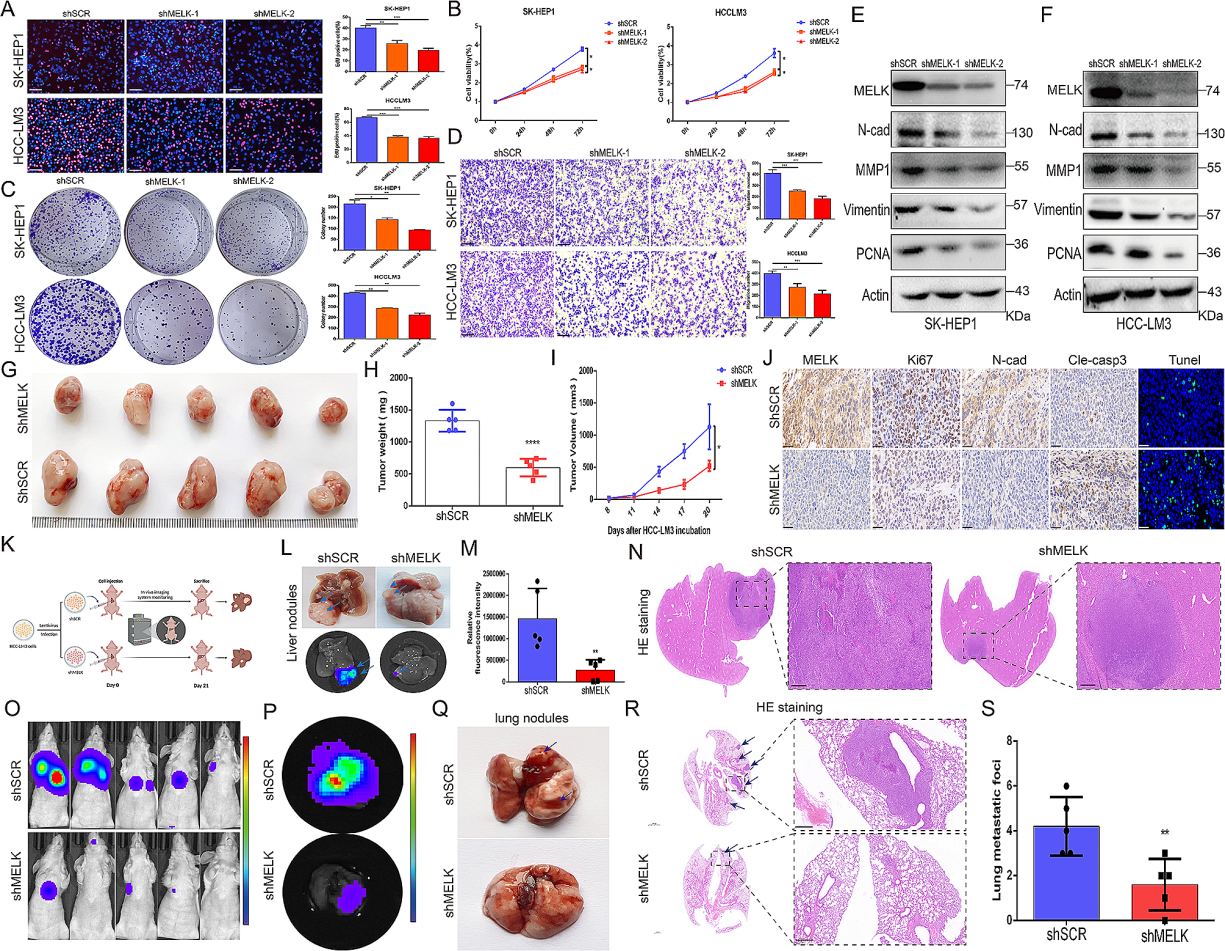
MELK contributes to the tumorigenesis, progression and spontaneous lung metastasis of HCC. (**A**) EdU assay confirming that MELK knockdown suppressed the proliferation of HCC cells. **(B)** CCK-8 assay showing the inhibition of MELK knockdown in the proliferation of SK-HEP1 and HCC-LM3 cells. **(C)** Colony formation assay showing the suppression of colony formation by MELK knockdown in HCC cells. **(D)** Transwell migration assay indicating that MELK knockdown impaired the migration of HCC cells. **(E-F)** WB assay showing the effect of MELK inhibition on the expression of proliferation- and migration-related factors. **(G)** Difference in the growth of HCC-LM3 tumors with or without MELK knockdown (*n* = 5/group). **(H-I)** Differences in tumor weight (H) and tumor volume (I) in HCC-LM3 tumors with or without MELK knockdown (*n* = 5/group). (J) IHC staining and TUNEL staining showing the effect of MELK inhibition on tumor proliferation, migration and apoptosis. **(K)** Construction scheme for the orthotopic implantation model of HCC-LM3 tumors in BALB/c nude mice. **(L)** General visualization and fluorescence imaging showing the difference in HCC-LM3 tumor growth with or without MELK knockdown. **(M)** The difference in the fluorescence intensity of HCC-LM3 tumors with or without MELK knockdown (*n* = 5/group). **(N)** HE staining reflecting pathological differences in tumor progression upon HCC-LM3 MELK knockdown. **(O-P)** In vivo bioluminescence imaging showing the inhibition of HCC-LM3 MELK in lung metastasis (*n* = 5/group). **(Q)** Macroscopic changes in lung metastasis upon HCC-LM3 MELK knockdown. **(R)** HE staining showing pathological changes in lung metastasis upon HCC-LM3 MELK knockdown. **(S)** Changes in intrapulmonary metastasis numbers upon HCC-LM3 MELK inhibition (*n* = 5/group). * *p* < 0.05, ** *p* < 0.01, *** *p* < 0.001, **** *p* < 0.0001

To elucidate the functional consequences of MELK upregulation in HCC, we used a combination of in vitro and in vivo studies. We first generated stable MELK knockdown SK-HEP1 and HCC-LM3 cells, and as determined by EdU assays and CCK-8 assays, knockdown of MELK significantly reduced HCC cell proliferation (Fig. 2A-B). Colony formation assays revealed that the colony numbers of MELK-inhibited HCC cells were significantly reduced compared to the colony numbers of the corresponding control cells (Fig. 2C). We also designed and synthesized MELK specific single guide RNA (sgMELK) with CRISPR/Cas9 system, and Figure S2A-B confirmed that the MELK was knockout in HCC cells. And consistently, CRISPR/Cas9-mediated MELK knockout substantially impaired the colony formation ability of HCC cells (Figure S2C). Furthermore, MELK inhibition effectively attenuated the migration of HCC cells (Fig. 2D). Compared with the corresponding control cells, MELK knockdown HCC cells presented significantly decreased expression of proliferation- and migration-related markers, including PCNA, vimentin and N-cadherin (Fig. 2E-F), and this consistent result was further validated by IF analysis (Figure S3A-H). The above results revealed that MELK plays a positive role in facilitating the proliferation and migration of HCC cells.

We then turned to in vivo loss-of-function experiments using MELK knockdown HCC-LM3 cell lines. We constructed BALB/c nude mouse tumor xenograft models by subcutaneously injecting HCC-LM3 cells transfected with shSCR or shMELK and found that MELK inhibition significantly suppressed tumor growth (Fig. 2G-I), confirming the pro-oncogenic effect of MELK. In addition, compared to the corresponding control tumors, MELK knockdown tumors presented reduced expression of Ki67 and N-cadherin, while cell apoptosis was significantly enhanced (Fig. 2J and S4A-E). Orthotopic implantation models of HCC-LM3 tumors in BALB/c nude mice were also constructed to further confirm the role of MELK in tumorigenesis and progression (Fig. 2K). Consistently, as indicated by macroscopic changes and pathological features, MELK knockdown substantially attenuated tumor occurrence and suppressed tumor growth (Fig. 2L-N). We also further evaluated the functional role of MELK in spontaneous lung metastasis through caudal vein injection of HCC-LM3 cells. The results showed that control HCC-LM3 cells caused more aggressive tumor lung metastasis than MELK knockdown HCC-LM3 cells (Fig. 2O-P). Figure 2Q also shows that MELK knockdown resulted in much slighter macroscopically visible metastases in the lung, and the attenuated metastasis capability was also determined by HE staining (Fig. 2R). MELK knockdown HCC-LM3 cells formed significantly fewer intrapulmonary metastases than the corresponding control HCC-LM3 cells (Fig. 2S). Collectively, these results support the conclusion that MELK is essential for the tumorigenesis, progression and metastasis of HCC.

### Mir-505-3p directly regulates MELK expression and inhibits HCC tumorigenesis

**Fig. 3:**
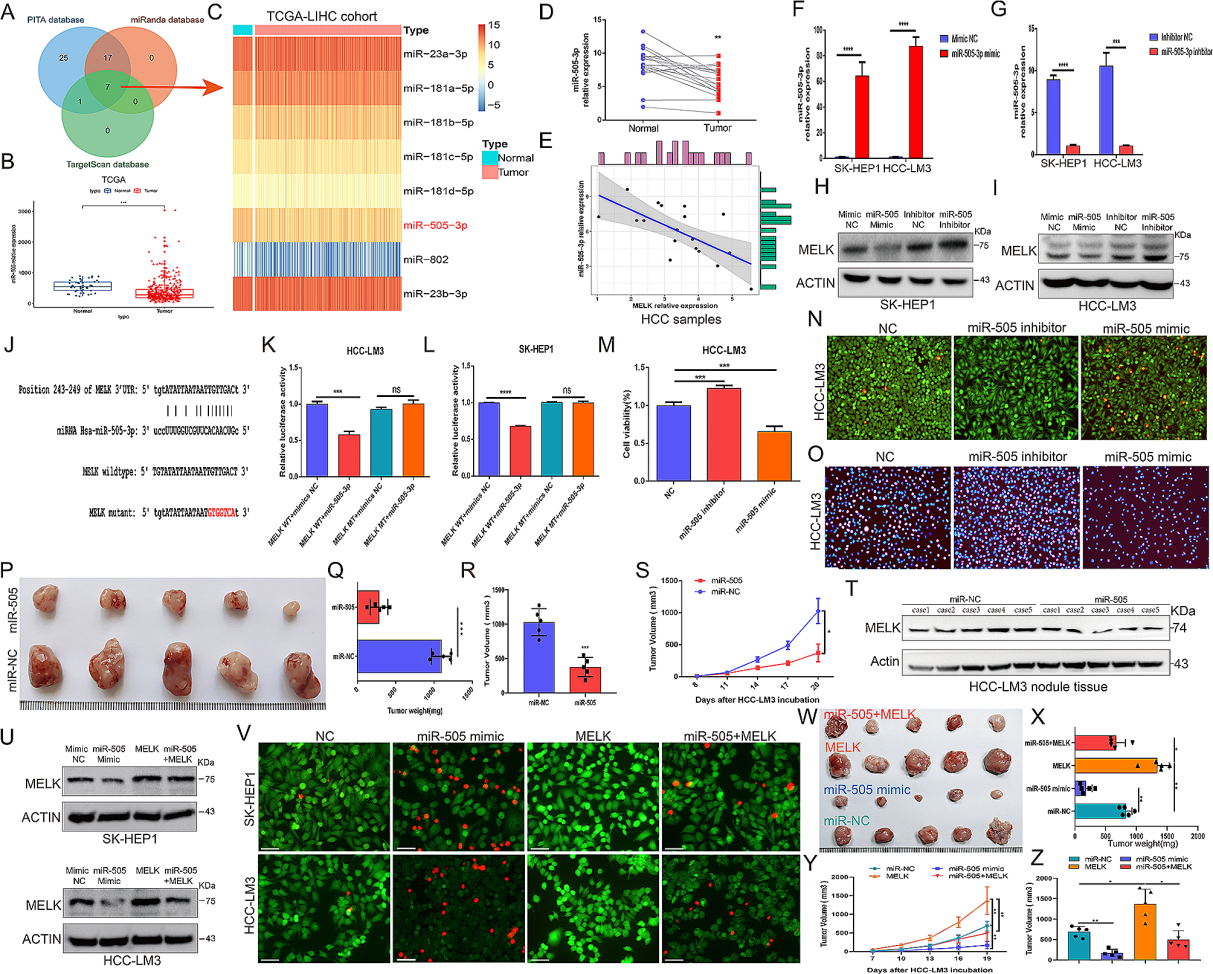
miR-505-3p serves as an upstream factor to target regulate the MELK-mediated tumorigenesis. (**A**) Prediction of potential upstream miRNAs regulating MELK using the PITA, miRanda and TargetScan databases. **(B-C)** Expression characteristics of the candidate miRNAs in the TCGA-LIHC cohort. **(D)** Differences in miR-505-3p expression in tumor tissues and corresponding samples in the validation cohort. **(E)** miR-505-3p expression showed a negative correlation with MELK expression in HCC samples. **(F-G)** Changes in the expression of miR-505-3p in HCC cells under miR-505-3p mimic (F) or miR-505-3p inhibitor transfection (G). **(H-I)** WB assay showing the effect of miR-505-3p changes on MELK expression in SK-HEP1 (H) and HCC-LM3 (I) cells. **(J)** Mutation strategy of predicted miRNA-targeting sites of miR-505-3p on the 3’-UTR of MELK. **(K-L)** Dual-luciferase reporter analysis confirming the miRNA-targeting sites of miR-505-3p on the 3’-UTR of MELK. **(M)** Effect of miR-505-3p expression on the viability of HCC-LM3 cells. **(N)** Live/dead cell assays showing the role of miR-505-3p in HCC-LM3 cell viability. **(O)** EdU assay showing the proliferation capability of HCC-LM3 upon miR-505-3p expression change. **(P)** Effect of miR-505-3p on the growth of HCC-LM3 tumors. **(Q-S)** Differences in tumor weight (Q) and tumor volume (R-S) in HCC-LM3 tumors in response to miR-NC and miR-505-3p treatment. **(T)** WB reflecting the change of MELK expression in response to miR-505-3p treatment. **(U)** WB showing the expression characteristic of MELK in response to the indicated treatments. **(V)** Live/dead cell assays indicating the inhibition effect of miR-505 on cell viability is reversed by MELK overexpression. **(W)** Suppression effect of miR-505-3p on the growth of HCC-LM3 tumors is diminished by forced expression of MELK (*n* = 5/group). **(X-Z)** Differences in tumor weight (X) and tumor volume (Y-Z) in HCC-LM3 tumors in response to the indicated treatment (*n* = 5/group). ns, no significance, * *p* < 0.05, ** *p* < 0.01, *** *p* < 0.001, **** *p* < 0.0001

As described above, MELK accelerates HCC tumorigenesis and development, but the underlying mechanism remains unclear. Numerous studies have demonstrated that miRNAs are involved in a variety of carcinogenic processes by binding to the 3’ untranslated region (3’UTR) of targeted mRNAs, giving rise to mRNA degradation or translation suppression [30]. Based on this, we explored the potential upstream miRNAs regulating MELK using multiple target-predicting programs (Fig. 3A) (Table S2) and identified eight candidate miRNAs (Fig. 3C). We then analyzed the expression difference of the candidate miRNAs in HCC tumor tissues and normal tissues from the TCGA database and unveiled substantially reduced expression of miR-505-3p in tumor tissues compared to normal tissues (Fig. 3B), and a consistent result was obtained in our collective HCC samples (Fig. 3D). Furthermore, we noted that MELK expression was negatively correlated with miR-505-3p expression (Fig. 3E). To further explore the relationship between MELK and miR-505-3p in HCC, we synthesized a miR-505-3p mimic to increase its expression level in HCC cells (Fig. 3F) and used a miR-505-3p inhibitor to effectively reduce the expression of miR-505-3p (Fig. 3G). As determined by WB, the miR-505-3p mimic significantly downregulated MELK, while the miR-505-3p inhibitor led to significant upregulation of MELK in HCC cells (Fig. 3H-I).

To better corroborate whether MELK is a direct target gene of miR-505-3p, we identified the miRNA-targeting sites of miR-505-3p on the 3’-UTR of MELK using an online bioinformatics assay (Miranda, TargetScan) and then cloned wild-type (WT) or the miR-505-3p binding site mutated MELK 3’-UTR (denoted MELK MT) into the pmirGLO dual-luciferase reporter (Fig. 3J). Reporter activity analysis showed that the miR-505-3p mimic effectively reduced the luciferase activities of the MELK WT reporter but had no effect on the reporters with MELK MT in HCC cells (Fig. 3K-L), supporting that MELK indeed a direct target of miR-505-3p. We also analyzed the functional role of miR-505-3p in HCC tumorigenesis and confirmed that the viability and proliferation of HCC cells were significantly enhanced by the miR-505-3p inhibitor but markedly suppressed by the miR-505-3p mimic (Fig. 3M-O). Moreover, the antitumor role of miR-505-3p was also further validated in vivo experiments, in which miR-505-3p mimic treatment substantially suppressed the growth and progression of tumor (Fig. 3P-S), and the MELK expression was significantly downregulated in response to miR-505-3p mimic treatment (Fig. 3T). To further prove that the antitumor effect of miR-505-3p is achieved through targeted regulation of MELK, we first performed WB analysis and confirmed that the the suppression of miR-505-3p mimic on MELK was reversed by MELK overexpression (Fig. 3U). Figure 3V further determined that the inhibition effect of miR-505 on cell viability could be reversed by MELK overexpression. Consistent with this, in vivo experiments also confirmed that the antitumor effect of miR-505-3p was substantially diminished by forced expression of MELK (Fig. 3W-Z).

These findings highlighted that miR-505-3p targets and regulates MELK expression directly and that MELK-mediated promotion of HCC tumorigenesis is dependent on the negative regulation of miR-505-3p.

### Interaction with STAT3/CCL2 is required for MELK-mediated HCC occurrence and progression

**Fig. 4:**
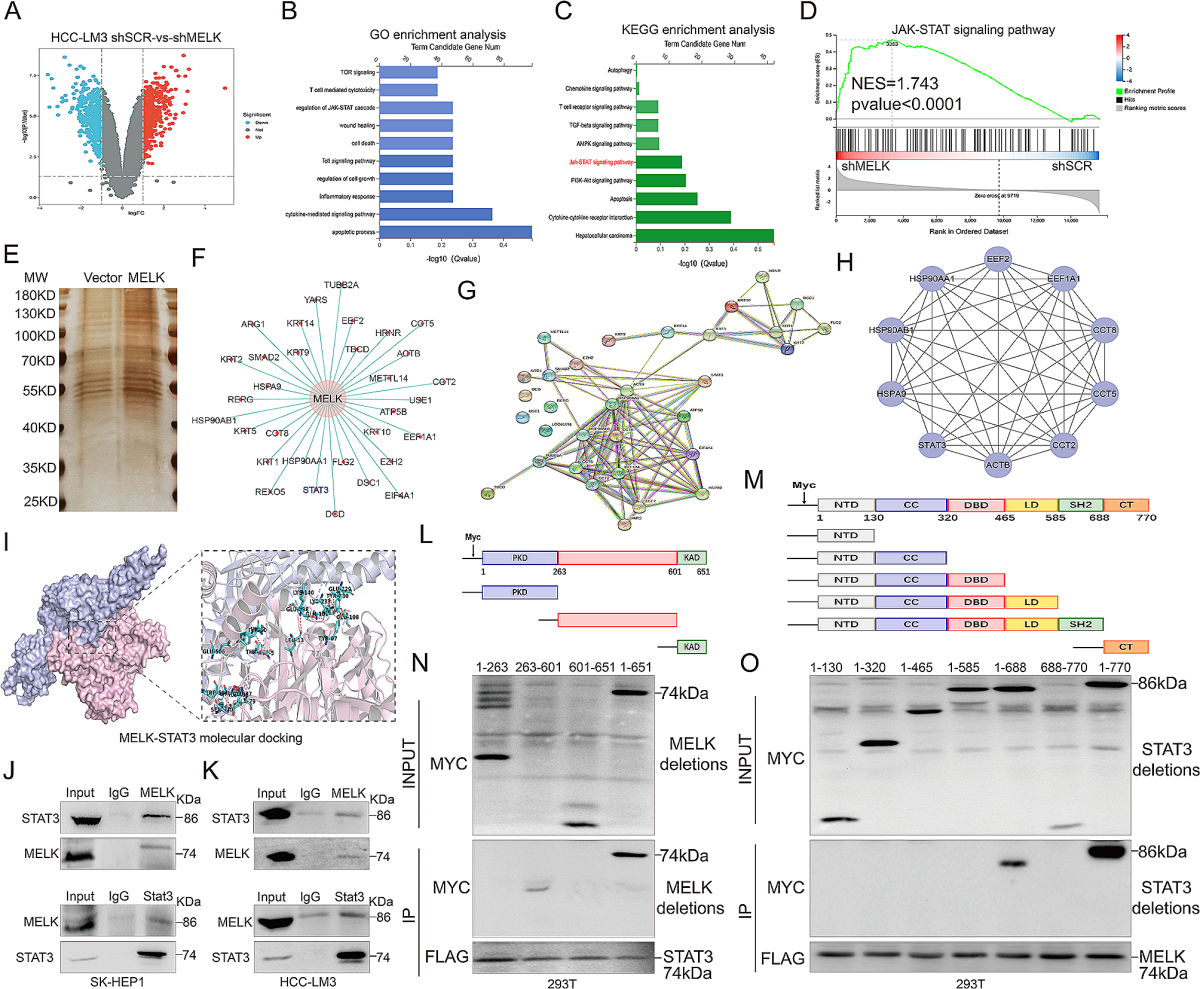
MELK interacts with STAT3 directly in HCC. (**A**) Volcano plot depicting the differentially expressed genes (DEGs) between MELK knockdown HCC-LM3 cells and the corresponding control HCC-LM3 cells. **(B)** GO enrichment analysis of the DEGs. **(C)** KEGG enrichment analysis of the DEGs. **(D)** GSEA revealing the close correlation between MELK expression and JAK-STAT signaling pathway activation. **(E)** Separation and visualization of the MELK-containing protein complex in 293T cells using SDS–PAGE and silver staining. **(F)** Mass spectrometry showing the proteins interacting with MELK in 293T cells. **(G)** PPI analysis for the 32 MELK-interacting proteins. **(H)** The hub 10 MELK-interacting proteins identified by PPI. **(I)** Molecular docking depicting the potential interaction sites of STAT3 and MELK. **(J-K)** Coimmunoprecipitation (CoIP) detecting the interaction between MELK and STAT3 in SK-HEP1 (J) and HCC-LM3 (K) cells. **(L)** Strategies to define different MELK fragments. **(M)** Strategies to define different STAT3 fragments. **(N)** Mapping the MELK fragment that interacts with STAT3. **(O)** Mapping the STAT3 fragment that interacts with MELK.

To further clarify the detailed downstream mechanism whereby MELK facilitates HCC occurrence and progression, we identified differentially expressed genes (DEGs) between MELK knockdown HCC-LM3 cells and the corresponding control HCC-LM3 cells (Fig. 4A). GO and KEGG enrichment analyses revealed that there was a close relationship between MELK-induced tumorigenesis and cell death-, immune response- and tumor signal transduction-related pathways such as “apoptosis”, “cytokine‒cytokine receptor interaction” and “JAK-STAT signaling pathway” (Fig. 4B-C), and GSEA further confirmed that MELK expression was a significant factor in JAK-STAT signaling pathway activation (Fig. 4D). To further identify specific factors correlated with MELK, 293T cells stably expressing FLAG-tagged MELK or empty vector were generated, and affinity purification and SDS–PAGE were performed to separate the cellular immunocomplexed proteins, followed by visualization by silver staining (Fig. 4E). Through liquid chromatography tandem mass spectrometry, we then detected 32 unique MELK-interacting proteins in FLAG-tagged MELK-expressing 293T cells in comparison to the vector-transfected cells (Fig. 4F). Performing the Protein‒protein interaction (PPI) analysis on the 32 proteins, we finally identified 10 hub MELK-interacting proteins (Fig. 4G-H). Considering the results of the gene functional enrichment analysis and mass spectrometry above, we finally supposed STAT3 as a potential crucial MELK-interacting protein, the result of molecular docking depicted the possible interaction sites of STAT3 and MELK (Fig. 4I), and the interaction between STAT3 and MELK was further validated via the CoIP analysis (Fig. 4J-K). We therefore proceeded to map the interacting domains in MELK and STAT3 and determined that STAT3 interacts with the linker domain of MELK (Fig. 4L and N), and MELK interacts with the STAT3 SH2 domain (Fig. 4M and O).

**Fig. 5:**
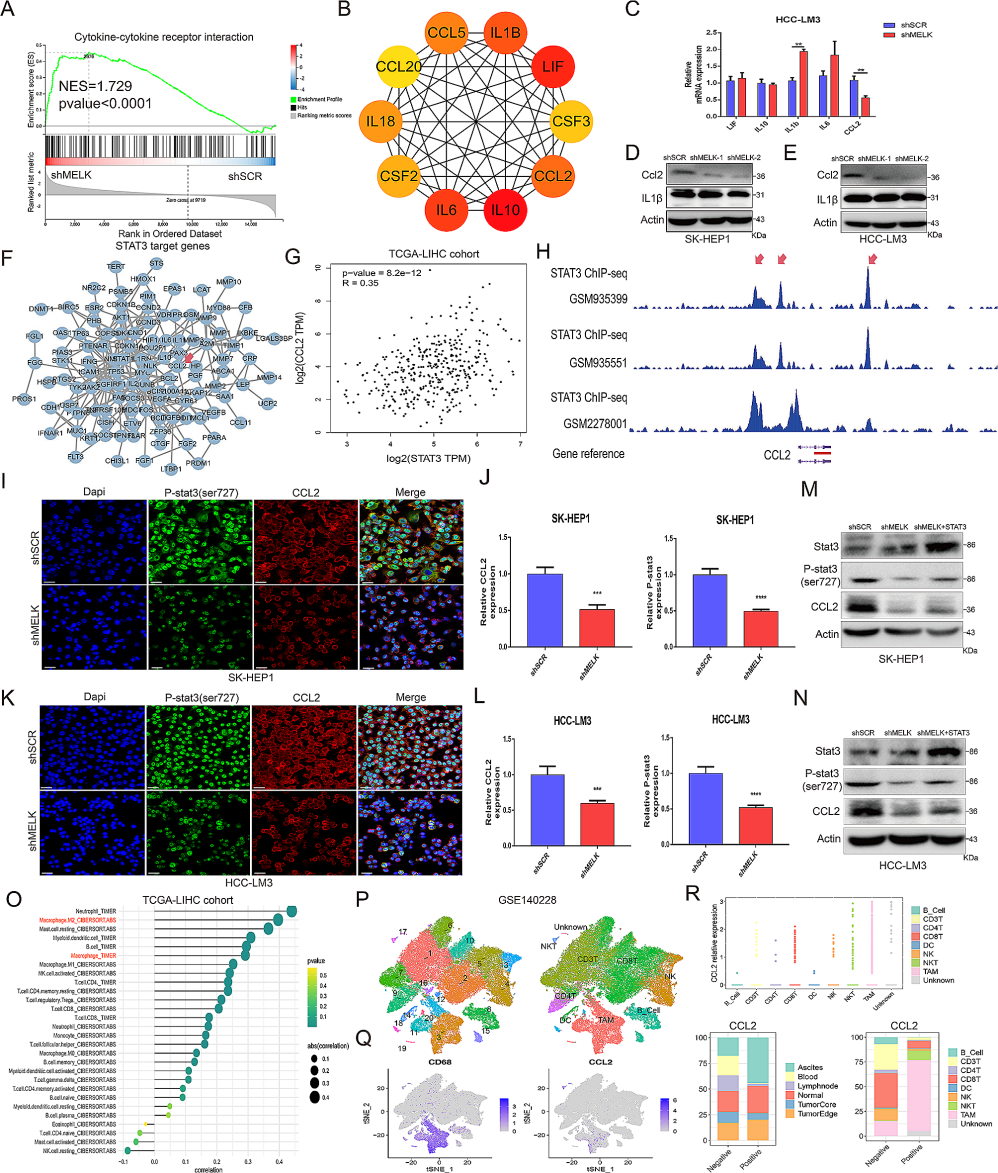
MELK activates STAT3 phosphorylation and increases the expression of its target gene CCL2 in HCC. (**A**) GSEA showing the close relationship between cytokine‒cytokine receptor interaction signal pathway activity and MELK expression in HCC-LM3 cells. **(B)** Protein‒protein interaction (PPI) analysis identifying 10 hub genes in the cytokine‒cytokine receptor interaction pathway closely related to MELK. **(C)** qRT‒PCR analysis detecting the effect of MELK on the expression of the identified hub genes in HCC-LM3 cells. **(D-E)** WB analysis showing the effect of MELK on the expression of CCL2 and IL-1β in SK-HEP1 (D) and HCC-LM3 cells (E). **(F)** STAT3 target genes predicted by the Gene Transcription Regulation Database (GTRD). **(G)** Correlation between the expression of STAT3 and CCL2 in the TCGA-LIHC cohort. **(H)** ChIP-seq assays showing the colocalization regions of STAT3 and CCL2. **(I-L)** IF staining reflecting the effect of MELK knockdown on the expression of phospho-STAT3 and CCL2 in SK-HEP1 (I-J) and HCC-LM3 cells (K-L). **(M-N)** WB analysis confirming that STAT3 overexpression reverses the MELK-mediated suppression of CCL2 expression in SK-HEP1 (M) and HCC-LM3 cells (N). **(O)** The correlation between CCL2 expression and immune cell infiltration in the TCGA-LIHC cohort. **(P)** The immune cellular landscape of HCC in the GSE140228 cohort. **(Q)** The expression profiles of CCL2 in different immune cells. **(R)** The correlation between CCL2 expression and the infiltration level of immune cells in tumors. ** *p* < 0.01, *** *p* < 0.001, **** *p* < 0.0001

Notably, our results of gene functional enrichment analysis (Fig. 4B-C) also indicated that MELK expression was strongly associated with immune-related pathways, among which cytokine‒cytokine receptor interaction signaling was the most relevant pathway (Fig. 5A). PPI analysis on the genes belonging to the cytokine‒cytokine receptor interaction pathway revealed 10 hub genes closely related to MELK (Fig. 5B), and subsequent qRT‒PCR and WB analysis further confirmed that MELK inhibition significantly reduced the expression of CCL2 in HCC cells (Fig. 5C-E). Moreover, when predicting the STAT3 target-regulated genes in the Gene Transcription Regulation Database (GTRD), we noted that CCL2 was also a potential target gene of STAT3 (Fig. 5F). The analysis results of the TCGA-LIHC cohort also indicated that there was a positive correlation between the expression levels of STAT3 and CCL2 (Fig. 5G). Thus, we used chromatin immunoprecipitation sequencing (ChIP-seq) analysis and revealed the colocalization region of STAT3 and CCL2 (Fig. 5H). The results of IF and WB showed that MELK inhibition significantly reduced the expression of phospho-STAT3 and CCL2 in HCC cells (Fig. 5I-L), while the inhibition was partially rescued by STAT3 overexpression (Fig. 5M-N). Notably, CCL2 is a strong chemokine that functions in recruiting monocytes and macrophages, so we analyzed the relationship between CCL2 expression and immune cell infiltration in the TCGA-LIHC cohort and found a close correlation between CCL2 and the infiltration of macrophages, especially M2 macrophages (Fig. 5O). Then, we used scRNA sequencing to further analyze the immune cellular landscape of HCC in the GSE140228 cohort (Fig. 5P) and unveiled the enhanced expression of CCL2 in CD68 + macrophages (Fig. 5Q). We also analyzed the correlation between the expression of CCL2 in tumor cells and macrophage infiltration in GSE125449 cohort and found that there was a positive correlation between the expression of CCL2 in KRT18 + tumor cells and CD68 + macrophages infiltration (Figure S5A-C). Assessing the expression characteristics of CCL2 in immune cells and different tissues, we found that CCL2 was strongly expressed in TAMs and exerted a substantial positive impact on the infiltration of TAMs while negatively associated with the infiltration of B cells and T cells (Fig. 5R). Further exploration on the relationship between CCL2 and TAMs as well as Cytotoxic T Lymphocyte (CTL) function unveiled that CCL2 was closely associated with the infiltration of TAMs including interferon-primed TAMs (IFN TAMs) and CD8 + T cells including proliferated CD8 + T cells and cytokines cyiotoxic CD8 + T cells (Figure S5D-E). Cell communication between TAMs and other cells, and cell communication between CD8 + T cells and other cells was shown in Figure S5F and Figure S5G implied that there is a close association between TAMs and CTL infiltration. Overall, these findings consolidated that STAT3 serves as a crucial MELK-interacting protein and that the MELK-mediated STAT3/CCL2 signaling axis accelerates HCC occurrence and progression; in addition, CCL2 is a noteworthy factor related to TAMs infiltration.

### Tumor cell-intrinsic MELK inhibition functions in interfering with the infiltration and polarization of TAMs in HCC

**Fig. 6:**
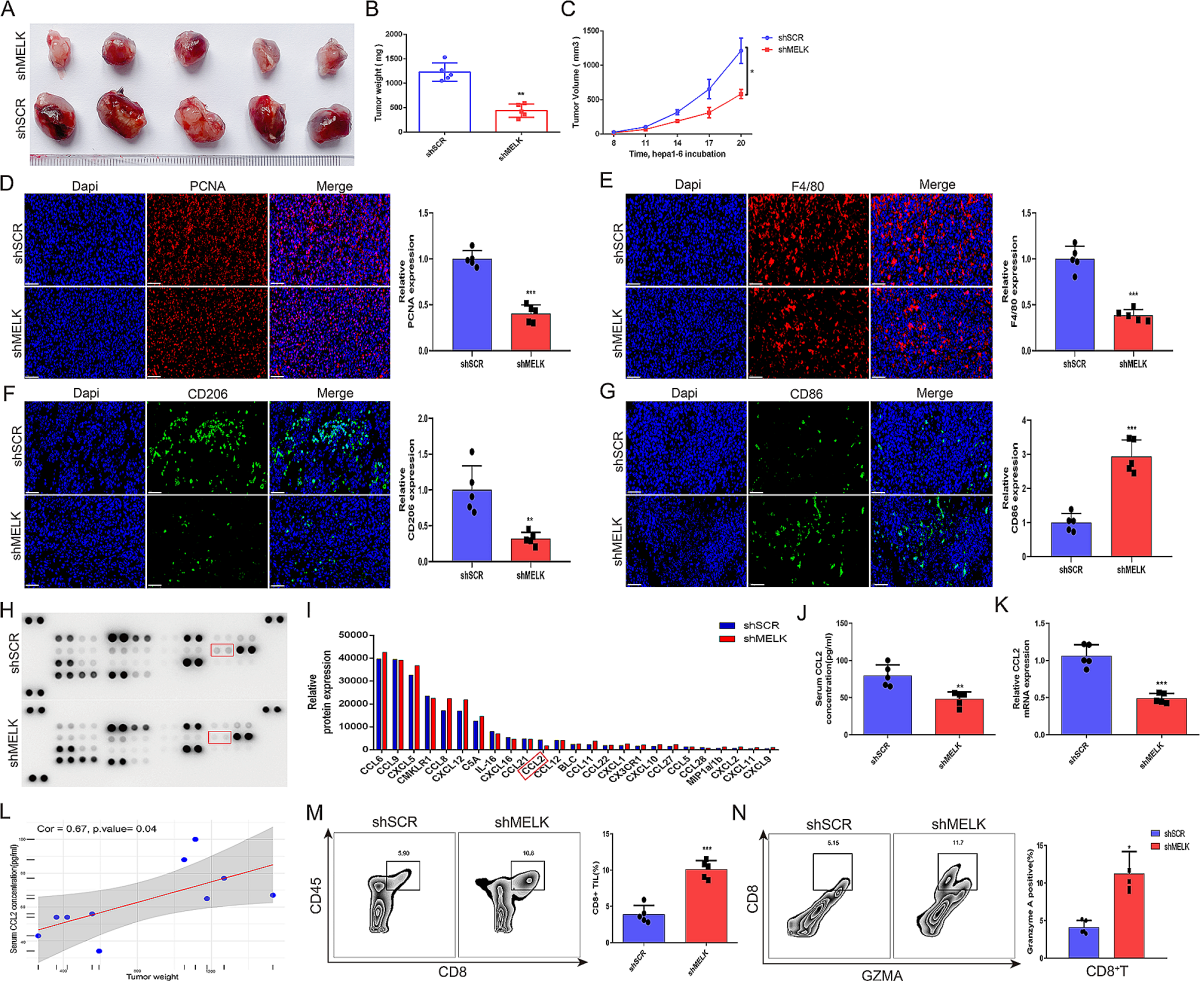
Tumor cell-intrinsic MELK inhibition affects the infiltration and polarization of TAMs. (**A**) The effect of MELK knockdown on tumor growth in mouse xenograft models (*n* = 5/group). **(B-C)** The effect of MELK inhibition on tumor weight (B) and tumor volume (C) (*n* = 5/group). **(D-G)** IF staining reflecting the expression changes of PCNA (D), F4/80 (E), CD206 (F) and CD86 (G) upon MELK inhibition in tumor tissues. **(H-I)** Antibody filter array implying the effects of MELK expression on cytokines related to immune cell recruitment. **(J)** ELISA showing the effect of tumoral MELK inhibition on serum CCL2 concentration. **(K)** qRT‒PCR analysis detecting the expression change of CCL2 upon MELK inhibition. **(L)** The correlation between tumor weight and serum CCL2 concentration. **(M)** FCM analysis showing the role of MELK expression in CD8 + T-cell recruitment to tumor tissue. **(N)** FCM analysis showing the effect of MELK expression on the GZMA-positive cell proportion in recruited CD8 + T cells in tumor tissue. * *p* < 0.05, ** *p* < 0.01, *** *p* < 0.001

The TME, with complex components and regulatory mechanisms, is regarded as the pivotal “fertile soil” supporting the occurrence and progression of tumors, in which TAMs, as the most important component of tumor-infiltrating immune cells, play an especially pivotal role in tumor development and antitumor immunity [31, 32]. Notably, our data implied that there was an association between MELK-mediated CCL2 expression and TAM infiltration (Fig. 5O-R and S4), which raised our interest in investigating the infiltration and functional alterations of macrophages upon tumoral MELK inhibition. MELK knockdown in Hepa1-6 cells significantly attenuated tumor growth in mouse xenograft models (Fig. 6A-C). The results of the IF assay suggested that compared to the control, MELK inhibition efficiently suppresses the expression of PCNA; notably, decreased expression of F4/80 and CD206 but increased expression of CD86 was observed in MELK-inhibited tumors (Fig. 6D-G), indicating that MELK inhibition may inhibit TAM infiltration, especially hampering protumoral M2 phenotype polarization but inducing M1 polarization.

To answer what factors in the TME affect the infiltration and polarization of TAMs upon tumoral MELK inhibition, we used an antibody filter array to survey the effects of MELK on cytokines related to immune cell recruitment and found that MELK knockdown led to a reduction in CCL2 production (Fig. 6H-I). We then performed ELISA to detect the secretion level of CCL2 in serum from mouse xenograft models bearing MELK knockdown tumors or the corresponding control tumors, and a reduced concentration of CCL2 was detected in MELK knockdown tumor-bearing mice (Fig. 6J). This consistent result was also confirmed by qRT‒PCR analysis (Fig. 6K). In addition, we noted that there was a positive correlation between tumor weight and serum CCL2 concentration (Fig. 6L). Interestingly and notably, in addition to the reduction in CCL2, the antibody filter array also showed that MELK knockdown upregulated CXCL9, CXCL10 and CXCL11, which are known to function in recruiting Th1, Th17 and cytotoxic T cells (also known as CD8^+^ T cells) [33]. We thus hypothesized that MELK may also be involved in the regulation of CD8^+^ T-cell recruitment. Consistent with our speculation, an increased proportion of CD8^+^ tumor-infiltrating lymphocytes (TILs), especially granzyme A (GZMA)-positive cells, was detected in MELK knockdown tumors (Fig. 6M-N). To sum up the above findings, we reasonably propose that MELK knockdown not only inhibits the infiltration and interferes with the polarization of TAMs, which is related to the expression of CCL2, but also strengthens the recruitment of CD8^+^ T cells to tumors.

### TAM functional alteration relies on CCL2 changes in the context of tumor cell-intrinsic MELK

**Fig. 7:**
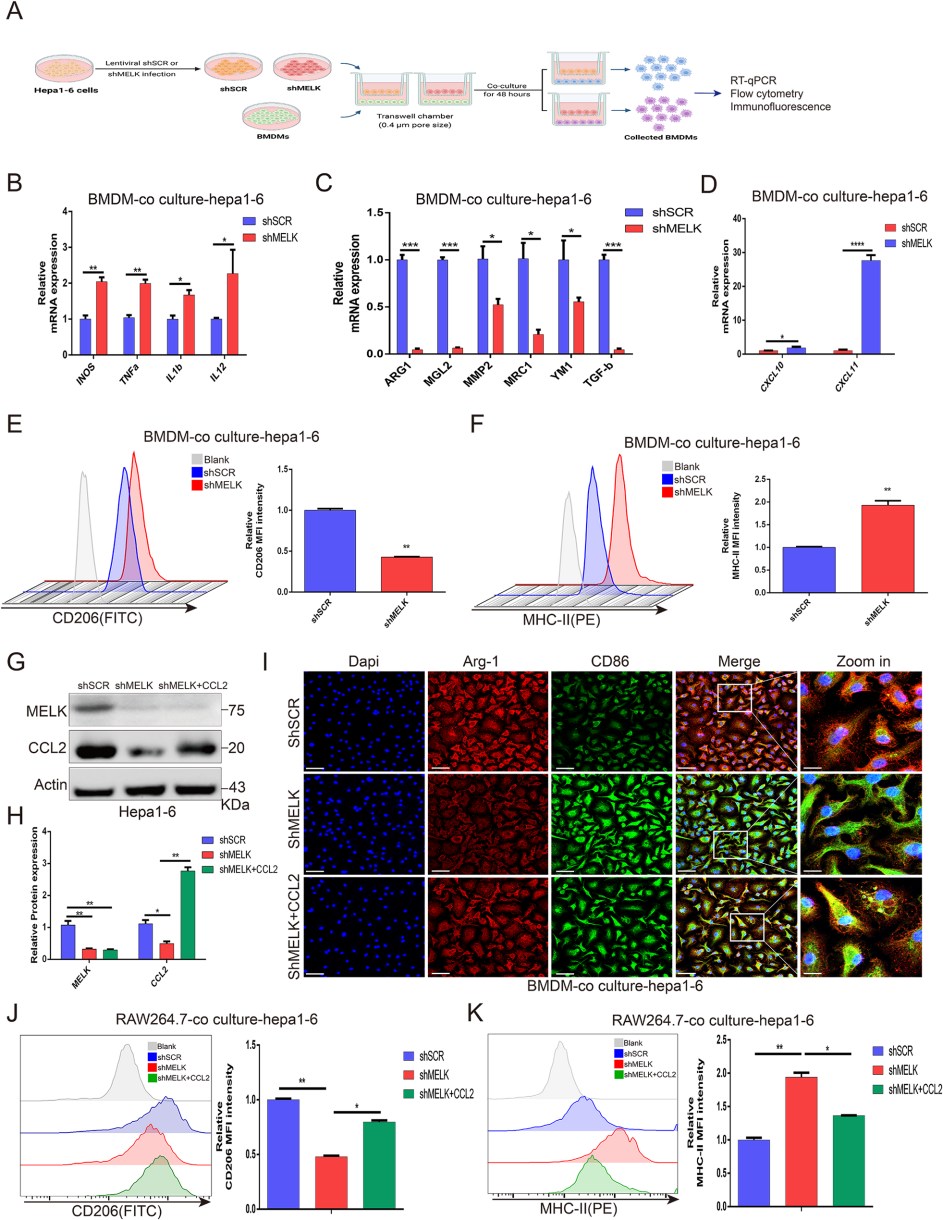
CCL2 is required for tumoral MELK-mediated TAM polarization in HCC. (**A**) Schematic illustration of the coculture system construction (drawn by https://biorender.com/). **(B-C)** qRT‒PCR revealing the expression changes in M1- and M2-related markers in BMDMs upon coculture Hepa 1–6 MELK inhibition. **(D)** qRT‒PCR showing the expression of CXCL10 and CXCL11 in BMDMs cocultured with MELK knockdown Hepa1-6 cells. **(E-F)** FCM analysis showing the expression changes of CD206 (E) and MHC-II (F) in BMDMs cocultured with MELK knockdown Hepa1-6 cells. **(G-H)** WB analysis confirming the expression changes in MELK and CCL2 in Hepa1-6 cells under different treatments. **(I)** IF staining reflecting the expression changes in ARG-1 and CD86 in BMDMs after coculturing with Hepa1-6 cells with different treatments. **(J-K)** FCM analysis detecting the expression characteristics of CD206 (J) and MHC-II (K) in RAW264.7 cells cocultured with Hepa1-6 cells under different treatments. * *p* < 0.05, ** *p* < 0.01, *** *p* < 0.001, **** *p* < 0.0001

To further investigate and characterize the functional link between CCL2 and TAMs, we established a coculture system and detected the polarization trend of BMDMs cocultured with Hepa1-6 cells with or without MELK knockdown (Fig. 7A). The qRT‒PCR results revealed that the MELK-deficient Hepa1-6 cell coculture system significantly inhibited M2 polarization while promoting M1 polarization (Fig. 7B-C), and the expression of CXCL10 and CXCL11 was substantially increased in BMDMs cocultured with MELK-deficient Hepa1-6 cells (Fig. 7D). In addition, a flow cytometry (FCM) assay also revealed the reduced M2 phenotype but enhanced M1 polarization (Fig. 7E-F). Then, we treated Hepa1-6 cells with MELK knockdown or the combination of MELK knockdown and CCL2 overexpression (Fig. 7G-H). We noted that the MELK knockdown-mediated reduction in M2 polarization and increase in M1 polarization in Hepa1-6 cells could be significantly reversed by CCL2 overexpression (Fig. 7I-K). Taken together, these data effectively confirmed the crucial role of CCL2 in MELK-mediated TAM polarization: MELK inhibition in HCC cells leads to the M2 reduction and M1 increase depending on the expression of CCL2.

### MELK inhibition enhances CCL2-mediated antitumor immunity and amplifies the antitumoral effect of RT

**Fig. 8:**
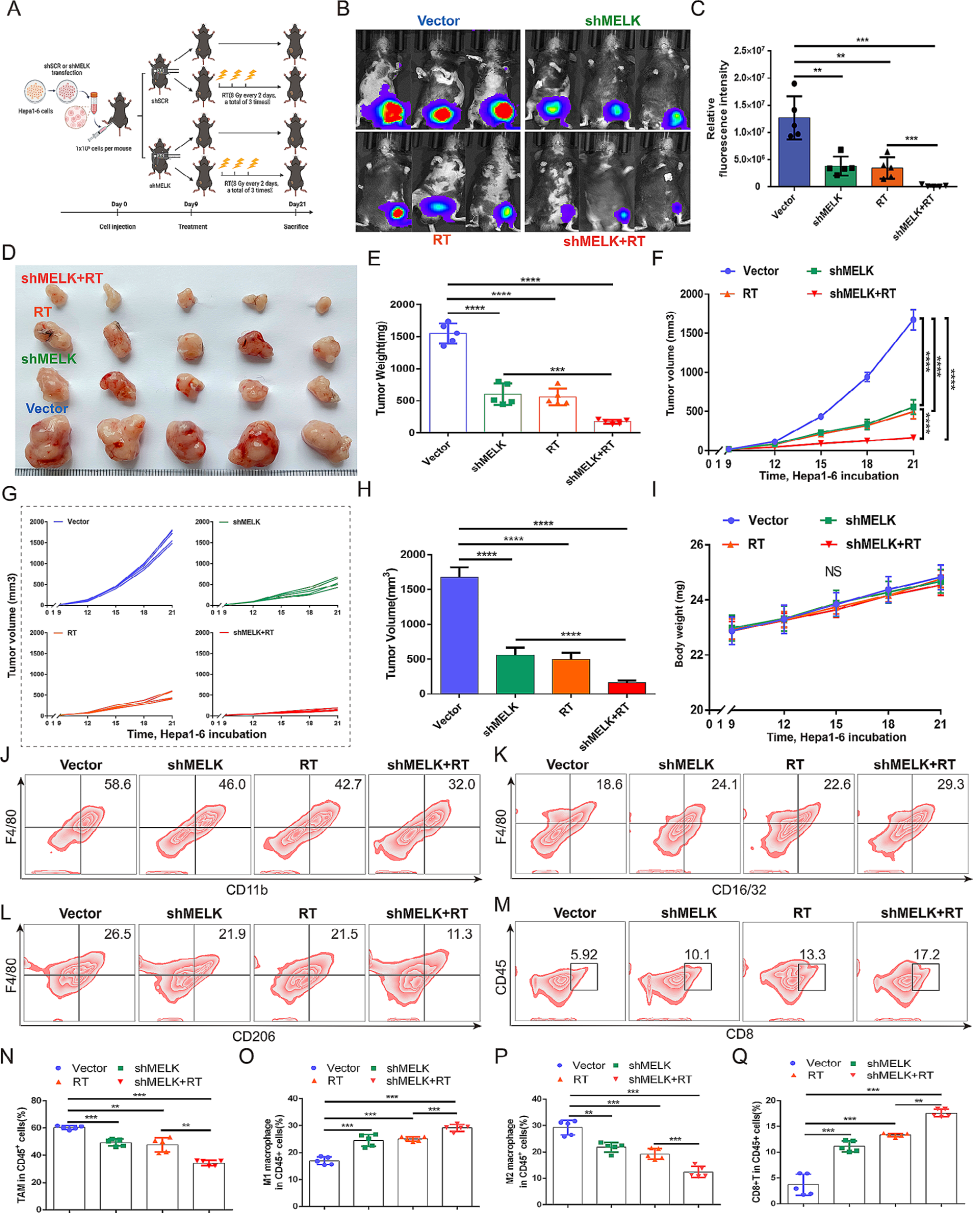
The combination of MELK knockdown and RT treatment achieves the optimal antitumoral effect compared to the single treatment. (**A**) Treatment scheme for Hepa1-6 tumor-bearing mouse models. **(B-C)** In vivo bioluminescence imaging showing subcutaneous Hepa1-6 tumors in mouse models. **(D)** Differences in tumor growth in response to different treatments (*n* = 5/group). **(E)** Differences in tumor weight in response to different treatments (*n* = 5/group). **(F-H)** Differences in tumor volume in response to different treatments (*n* = 5/group). **(I)** Body weight of mouse models in response to different treatments (*n* = 5/group). **(J-Q)** Differences in the infiltration level of TAMs (J, N), M1 phenotype (K, O), M2 phenotype (L, P) and CD8 + T cells (M, Q) in tumors in response to different treatments (*n* = 5/group). ** *p* < 0.01, *** *p* < 0.001, **** *p* < 0.0001

RT plays an effective anticancer role in HCC, which is mainly attributed to its ability to induce DNA damage and elicit specific immune responses, but its efficacy is also restricted by the limited scope of direct tumor killing [18]. Notably, evidence shows that significant elevation of MELK is exhibited in high-grade glioma (HGG) tumors following the failure of radiation and plays a crucial role in tumor radioresistance [34]. We thus focused on investigating whether there was also a correlation between the expression of MELK and the clinical efficacy of RT for HCC. Compared to that in primary untreated HCC, the expression of MELK was significantly increased in recurrent HCC following the failure of radiation (Figure S6A). By GSEA, we found that MELK plays pivotal roles in the activation of signaling pathways, including “DNA replication”, “mismatch repair” and “nucleotide excision repair” (Figure S6B-D), which are crucial pathways closely related to the clinical antitumor efficacy of RT. We then further evaluated whether combining RT with MELK knockdown in tumors could enhance the anticancer efficiency of RT treatment in subsequent experiments (Fig. 8A). As shown, the combination of MELK inhibition and RT achieved more robust tumor suppression than MELK inhibition or RT treatment alone (Fig. 8B-C).MELK inhibition or RT treatment alone effectively attenuated tumor growth; in comparison, the combination of MELK inhibition and RT treatment achieved optimal anticancer efficacy (Fig. 8D-H). There was no significant difference in the body weights of mice after the different treatments, ruling out the potential biotoxicity of MELK inhibition or RT treatment (Fig. 8I). These results consolidated that combining MELK knockdown with RT treatment is beneficial for enhancing the antitumoral effect.

Then, we further explored the role and underlying mechanism of immune microenvironment regulation in the combination of MELK knockdown with RT treatment, and we analyzed immune infiltration in tumor tissues. The results of the FCM assay indicated that compared with MELK knockdown or RT treatment alone, the combination of MELK knockdown with RT treatment led to the lowest infiltration of TAMs, and antitumoral M1 polarization was stimulated while M2 polarization was substantially restrained; the combined treatment induced much more CD8^+^ T-cell recruitment to tumor tissues (Fig. 8J-Q). Then, we cocultured BMDMs with Hepa1-6 cells to further validate the immunoregulatory role of MELK inhibition in enhancing RT efficiency (Figure S7A). Performing an IF assay, we found that compared to the single treatment, the combination of MELK knockdown and RT treatment further lowered the polarization of M2 and stimulated the production of M1 (Figure S7B), while the regulatory effect was partially reversed by CCL2 overexpression. Consistently, as shown in FCM, MELK knockdown or RT treatment effectively strengthened M1 polarization while suppressing M2 polarization, and the regulatory role in macrophage polarization got strongest with the combination of RT and MELK knockdown, while it was partially reversed by CCL2 overexpression (Figure S7C-F). In summary, these data implied that MELK knockdown is beneficial in enhancing the anticancer effect of RT, which was achieved by regulating CCL2-mediated infiltration and functional alteration of immune cells, thereby amplifying RT-related immune effects.

### Pharmacological inhibition of MELK in combination with RT treatment exerts substantial antitumoral effects on HCC

**Fig. 9:**
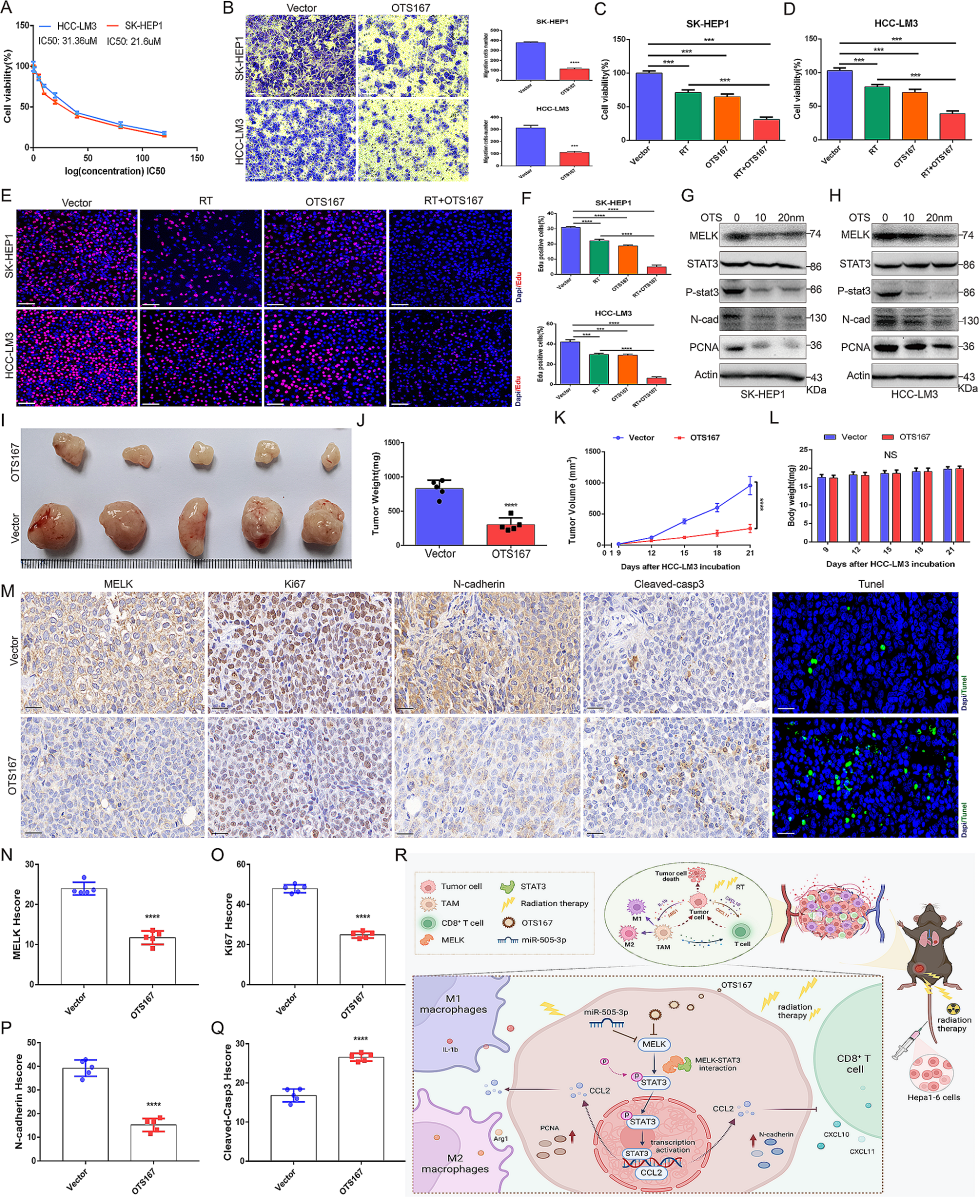
The combination of pharmacological inhibition of MELK and RT substantially restrains tumorigenesis and progression of HCC. (**A**) Confirmation of the half maximal inhibitory concentration (IC50) of OTS167 in HCC cells. **(B)** Transwell migration assays showing the effect of OT167 on the migration of HCC cells. **(C)** CCK-8 assays detecting the cell viability of SK-HEP1 (C) and HCC-LM3 cells (D) in response to different treatments. **(E-F)** EdU assays depicting the proliferation of HCC cells in response to different treatments. **(G-H)** WB analysis showing the expression of tumorigenesis- and progression-related factors in SK-HEP1 (G) and HC-LM3 cells (H) in response to different treatments. **(I-K)** The effect of OTS167 on Hepa1-6 tumor growth (I), tumor weight (J) and tumor volume (K) in mouse models (*n* = 5/group). **(L)** The effect of OTS167 on the body weight of mouse models (*n* = 5/group). **(M-Q)** IHC staining and TUNEL staining showing the expression of MELK, Ki67, N-cadherin, cleaved casp3 and apoptotic cells in tumor tissues under OTS167 treatment (*n* = 5/group). **(R)** Schematic diagram displaying the overall process and mechanism of this study (drawn by https://biorender.com/). ns, no significance, * *p* < 0.05, *** *p* < 0.001, **** *p* < 0.0001

We also extended our study to evaluate the functional role of pharmacological inhibition of MELK in regulating the progression of HCC. The half maximal inhibitory concentration (IC50) of OTS167 was verified to be 31.36 µM in HCC-LM3 and 21.6 µM in SK-HEP1 cells (Fig. 9A). We found that OTS167 treatment effectively restrained the migration of HCC cells (Fig. 9B), and the combination of RT and OTS167 treatment further decreased the viability and proliferation of HCC cells compared to that with RT or OTS167 treatment alone (Fig. 9C-F). WB analysis confirmed the dose-dependent inhibitory effect of OTS167 on phospho-STAT3, PCNA and N-cadherin (Fig. 9G-H). To further support the in vitro findings, the effect of OTS167 on tumor growth was also examined in vivo. As shown, OTS167 treatment significantly attenuated tumor growth compared to that with the control vector treatment (Fig. 9I-K), but there was no significant toxicity of OTS167 (Fig. 9L and S8). The results of IHC staining indicated that OTS167 treatment significantly inhibited the expression of MELK, Ki67 and N-cadherin while upregulating Cleaved-casp3, and TUNEL staining of tumor sections revealed an increased number of apoptotic tumor cells under OTS167 treatment (Fig. 9M-Q and S9). Overall, these findings elucidated that pharmacological inhibition of MELK effectively hampered the tumorigenesis and progression of HCC, and the combination with RT treatment is profited to strengthen the antitumoral effect. Figure 9R summarizes the overall process and mechanism of this study.

## Discussion

As one of the most prevalent and fatal malignant tumors, HCC seriously debilitates human health [35]. Due to an insufficient understanding of the complex molecular heterogeneity of hepatocarcinogenesis and the complex crosstalk between tumor cells and the TME, the effectiveness of existing treatment approaches remains limited, and the prognosis of patients is still not satisfactory [36, 37]. Hence, deciphering the key drivers of HCC tumorigenesis is crucial for exploiting novel targeted therapies aimed at the underlying cellular and molecular pathways. There is increased evidence for the hyperactivation of MELK in a broad range of human malignancies, where MELK plays a central role in tumorigenesis and is involved in tumor progression through multiple pathways [7, 8, 38]. However, the functional consequence of MELK in HCC tumorigenesis and the specific mechanism remains to be elucidated. In our study, we carried out a series of bioinformatic analyses on online databases, including TCGA, ICGC and GEO databases, and confirmed the prognostic role of MELK in predicting the poor outcome of HCC patients. Subsequent comprehensive in vitro and in vivo analyses further revealed the crucial effect of MELK expression on accelerating the occurrence, progression and metastasis of HCC. The pharmacological inhibition of MELK using OTS167 also led to an effective antitumor response.

To better corroborate the specific mechanism by which MELK facilities the progression of HCC, we predicted the potential upstream component targeting MELK using the PITA, miRanda and TargetScan databases. Using dual-luciferase reporter assays, we finally confirmed that miR-505-3p is the critical upstream factor regulating the expression of MELK by binding directly to the 3’ UTR of MELK. miR-505-3p has been reported as an effective tumor inhibitor in multiple cancer types. For example, miR-505-3p was shown to inhibit osteosarcoma tumorigenesis by targeting HMGB1 directly [39]. It was also reported that upregulation of miR-505-3p is involved in the reduced tumorigenicity of cervical cancer cells in vivo [40]. Consistent with previous findings, our data also determined the antitumor effect of miR-505-3p on HCC, which was achieved by its direct regulation of MELK expression, and the antitumor effect of miR-505-3p could be diminished by forced expression of MELK. In addition to the identification of the MELK upstream regulator miR-505-3p, we also substantiated the prominent role for the STAT3/CCL2 axis interaction in MELK-mediated HCC tumorigenesis and progression by combining bioinformatic analysis and cell experiments, including CoIP and ChIP assays. MELK was determined to bind and interact with the STAT3 SH2 domain directly, activate STAT3 phosphorylation and increase the expression of its target gene CCL2 in HCC. STAT3 is a member of the signal transducer and activator of transcription family, which mainly plays a role in a series of biological processes, including cell proliferation, survival, differentiation, and angiogenesis [41]. The function of STAT3 is dependent on the SH2 domain, which arbitrates multiple protein–protein interactions [42]. CCL2 was one of the first discovered chemokines synthesized and secreted mainly by monocytic cells [43]. Abundant evidence has confirmed that overexpression of CCL2 boosts tumor metastasis and invasion and induces immune resistance [44]. CCL2 is known to be a strong promoter of monocyte and macrophage recruitment, which regulates the tumor immune microenvironment [45]. Consistently, our data revealed a significant positive correlation between the infiltration of TAMs and CCL2 expression in HCC,, and what is also with noticing is that a negative correlation was revealed between CCL2 and immune cells including B cells, NK cells and T cells especially CD8 + T cells, which implies the role of CCL2 in tumor immune microenvironment and that inhibition of CCL2 may contribute to strengthening the antitumor immune response.

Given the strong chemotactic capability of CCL2 in recruiting monocytes and macrophages, we also broadened the functional link between tumoral MELK inhibition and immune cell activity in the TME. Notably, our findings provide a novel interaction atlas of tumor cells and the TME in MELK-deficient HCC. The abundance and composition of immune cells in the TME are significantly related to the progression of cancer and the effect of adopted therapeutic strategies [46]. Here, compared to that in the matched control Hepa1-6 tumors, the infiltration of TAMs was significantly reduced in MELK-inhibited Hepa1-6 tumors; in these tumors, the proportion of protumor- and immunosuppressive-related M2 phenotypes was especially reduced, while the proportion of M1 phenotype macrophages, which play a role in tumor suppression, was substantially elevated. In addition, a higher percentage of CD8 + T cells was detected in MELK-deficient Hepa1-6 tumors than in the corresponding control Hepa1-6 tumors, indicating that tumoral MELK inhibition induces the activation and recruitment of CD8 + T cells to the TME. CD8 + T cells are pivotal tumor cell-killing immune cells in the host antitumor immune response and responsible for disrupting tumor occurrence and metastasis via intracellular antigen-mediated tumor cell recognition and direct killing [47]. The number of CD8 + T cells is also considered to be an indicator of cancer regression [48]. In our subsequent analysis, we further revealed that the regulatory effect of MELK on immune cell activity in the TME was required for the expression of CCL2. Our study highlights complex and active crosstalk between HCC cells and the TME. As shown by our analyses, MELK inhibition conducted a substantial antitumor role in HCC, which may be partly achieved by CCL2-mediated reprogramming of the crosstalk between HCC cells and the TME to suppress TAM infiltration, interfere with TAM polarization and stimulate CD8 + T-cell recruitment, thereby strengthening antitumor immunity. The dichotomous roles of RT in the tumor immune microenvironment is a critical challenge to the efficiency of RT [49, 50]. To this end, we further evaluated the antitumor effect on HCC of the combination of RT and MELK exhaustion in tumors. We were pleased to find that MELK inhibition significantly strengthened the antitumor effect of RT, which was achieved by regulating CCL2-mediated infiltration and functional alteration of immune cells, thereby amplifying RT-related immune effects. The combination of RT and MELK exhaustion in tumors exerts an optimal therapeutic effect better than RT or MELK inhibition treatment alone.

Inevitably, there are some limitations in this study. Our study determined the pro-tumoral role of MELK in hepatocarcinogenesis and using CRISPR/Cas9-mediated MELK knockout system to confirmed the crucial role of MELK in the proliferation of HCC, but since most data in this study are based on shRNA-mediated MELK knockdown, which may have potential off-target effect. In the future following studies we plan to perform more CRISPR/Cas9-meidated gene knockout experiments and conduct transgenic mouse models to further verify the function of MELK in hepatocarcinogenesis. Besides, though there have been numerous published researches on OTS167 as a MELK inhibitor, there are also studies suggesting potential off target effects [51, 52]. Further research is still needed on the role and potential mechanism of OTS167 as a MELK inhibitor in HCC through more CRISPR/Cas9-meidated gene knockout experiments and transgenic mouse models. In addition, RT also has limitations as it is not suitable for advanced HCC patients with widely distributed intrahepatic lesions, Child Pugh C-grade liver function, or severe internal medicine underlying diseases in clinic. In future studies, we will further identify potential candidates suitable for RT combined with MELK inhibition.

## Conclusions

Taken together, these results highlight the clinical value of MELK as a promising therapeutic target in precise HCC therapy and indicate that OTS167 serves as an effective antagonist for HCC. MELK is regulated by miR-505-3p, interacts with STAT3, activates STAT3 phosphorylation and increases the expression of its target gene CCL2 in HCC. In addition, CCL2-induced TAM infiltration and functional alterations and CD8 + T-cell recruitment contribute to MELK-mediated HCC tumorigenesis and progression, and in particular, the regulatory role of MELK in the tumor immune microenvironment helps to amplify the RT-related immune effects to exert superior synergistic antitumor effects. Our analysis pinpoints promising targets for precise molecular therapy in HCC and provides an important step toward understanding and applying novel synergistic combination therapies.

### Electronic supplementary material

Below is the link to the electronic supplementary material.

Supplementary Material 1

Supplementary Material 2

Supplementary Material 3

Supplementary Material 4

Supplementary Material 5

Supplementary Material 6

Supplementary Material 7

Supplementary Material 8

Supplementary Material 9

Supplementary Material 10

Supplementary Material 11

Supplementary Material 12

Supplementary Material 13

## Data Availability

The data used to support the findings of this study are available from the corresponding author upon request. The RNAseq data analyzed in this study were accessed with the online databases including TCGA, ICGC, GEO, PITA, miRanda and TargetScan databases.
