# Supplementary figures and images for: Tumor cell-intrinsic MELK enhanced CCL2-dependent immunosuppression to exacerbate hepatocarcinogenesis and confer resistance of HCC to radiotherapy
Source: Mol Cancer. 2024 Jul 5;23:137. doi: 10.1186/s12943-024-02049-0 (PMC11225310; doi:10.1186/s12943-024-02049-0)

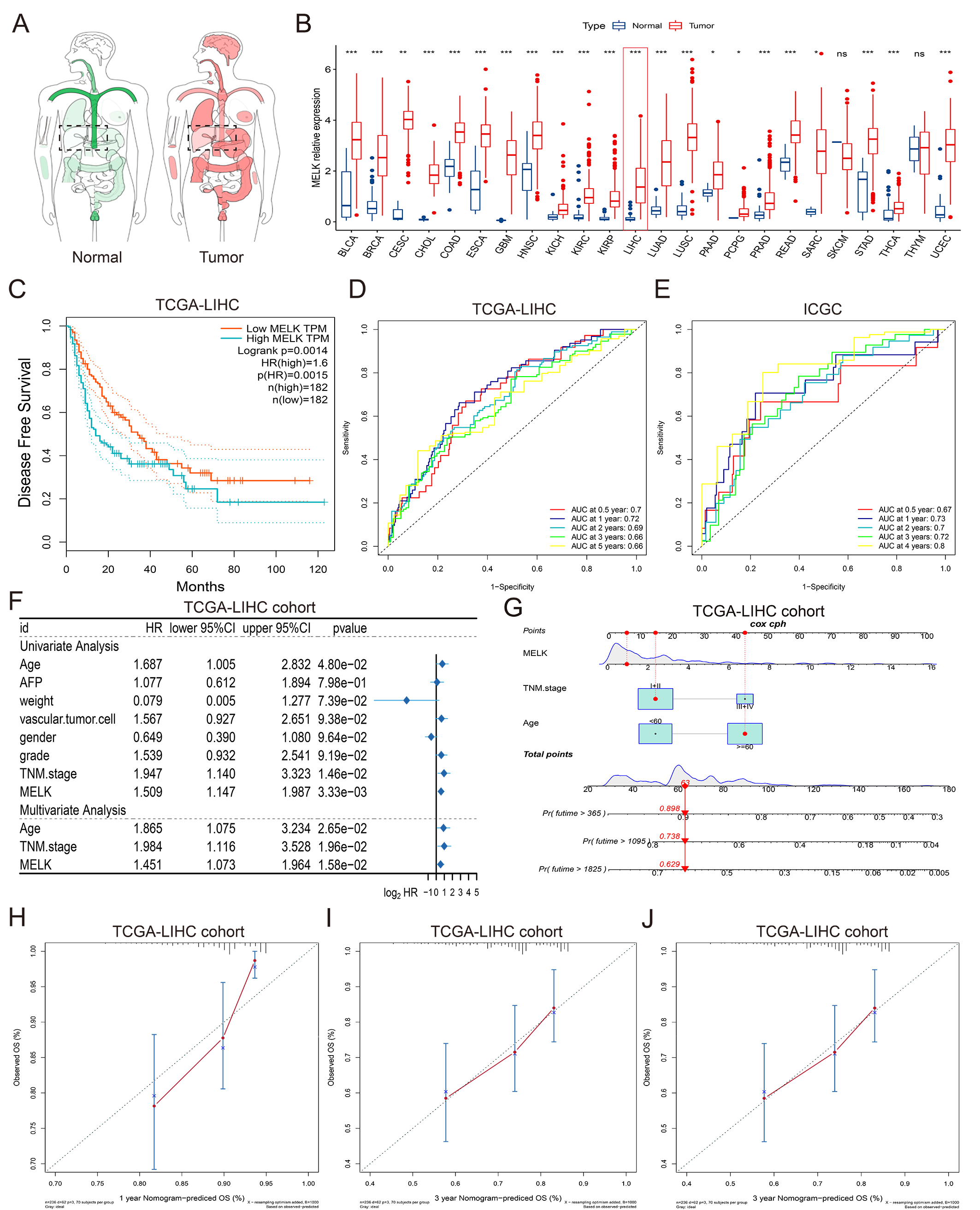

Supplement: Supplementary file 1 — Supplementary Material 1 [file 12943_2024_2049_MOESM1_ESM.tif]

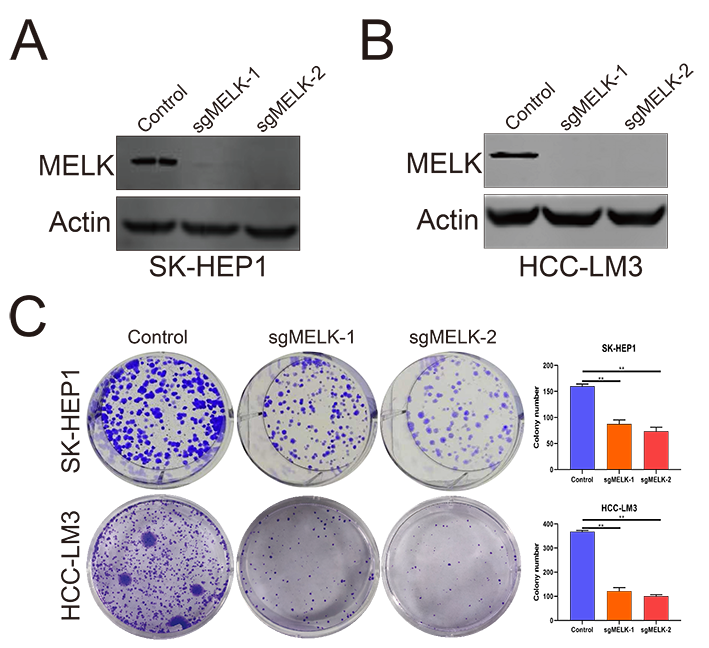

Supplement: Supplementary file 2 — Supplementary Material 2 [file 12943_2024_2049_MOESM2_ESM.tif]

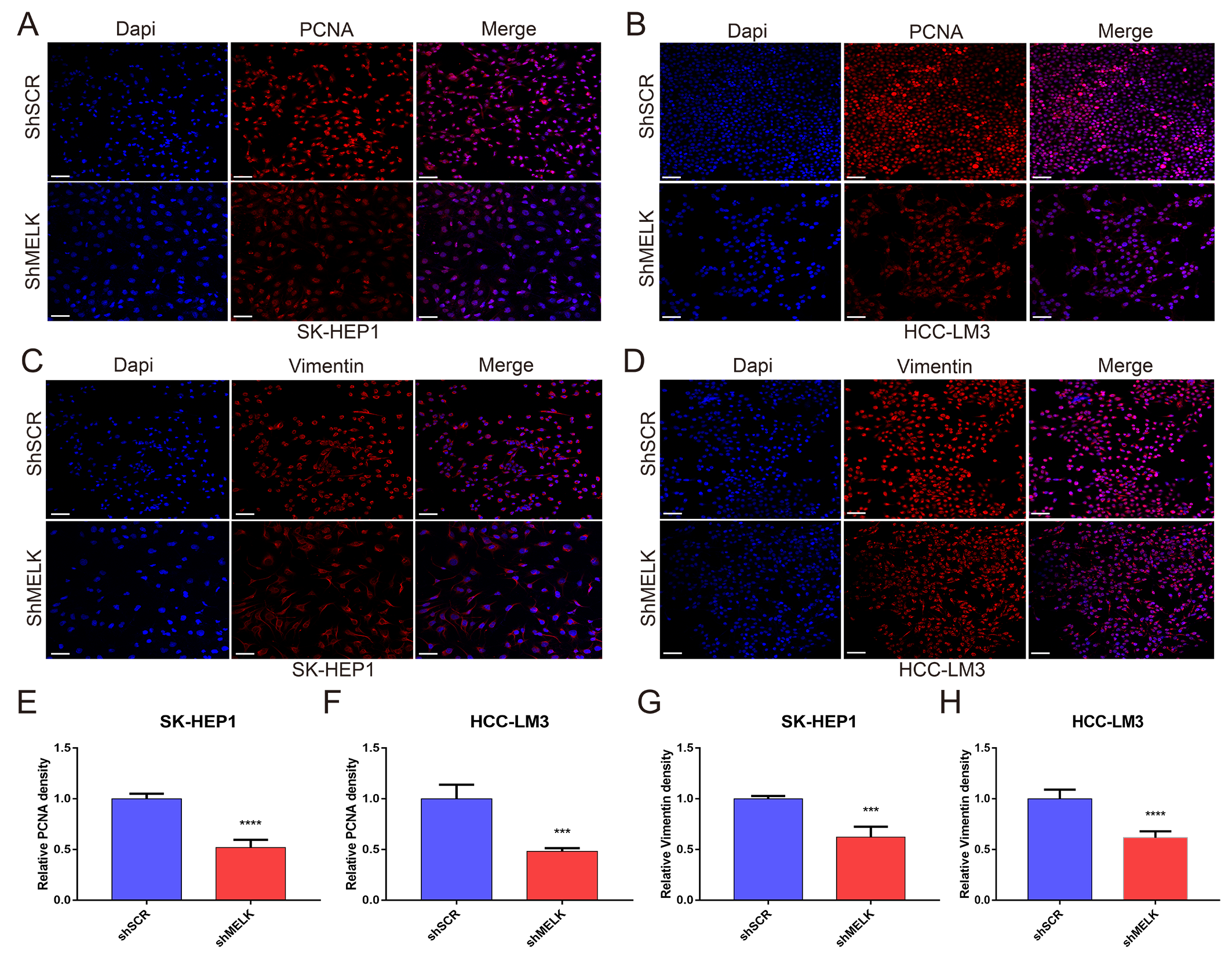

Supplement: Supplementary file 3 — Supplementary Material 3 [file 12943_2024_2049_MOESM3_ESM.tif]

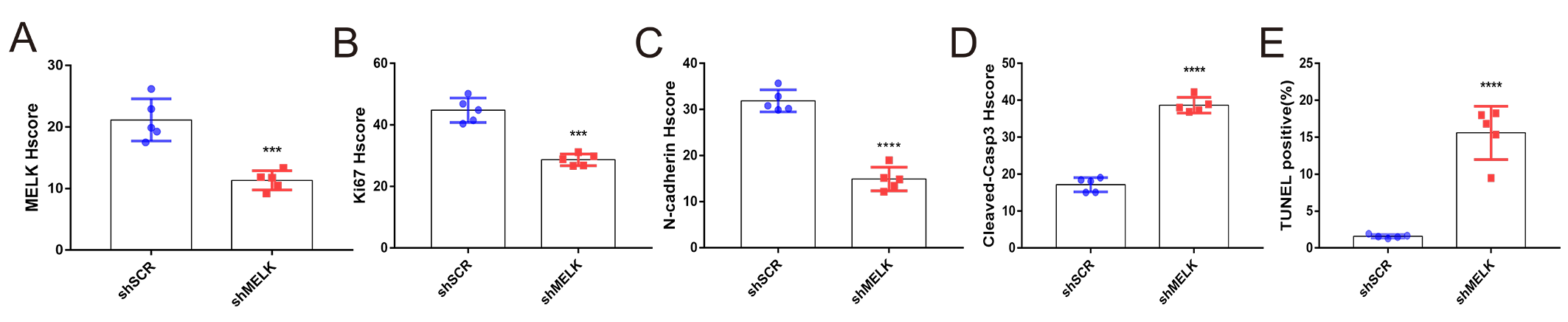

Supplement: Supplementary file 4 — Supplementary Material 4 [file 12943_2024_2049_MOESM4_ESM.tif]

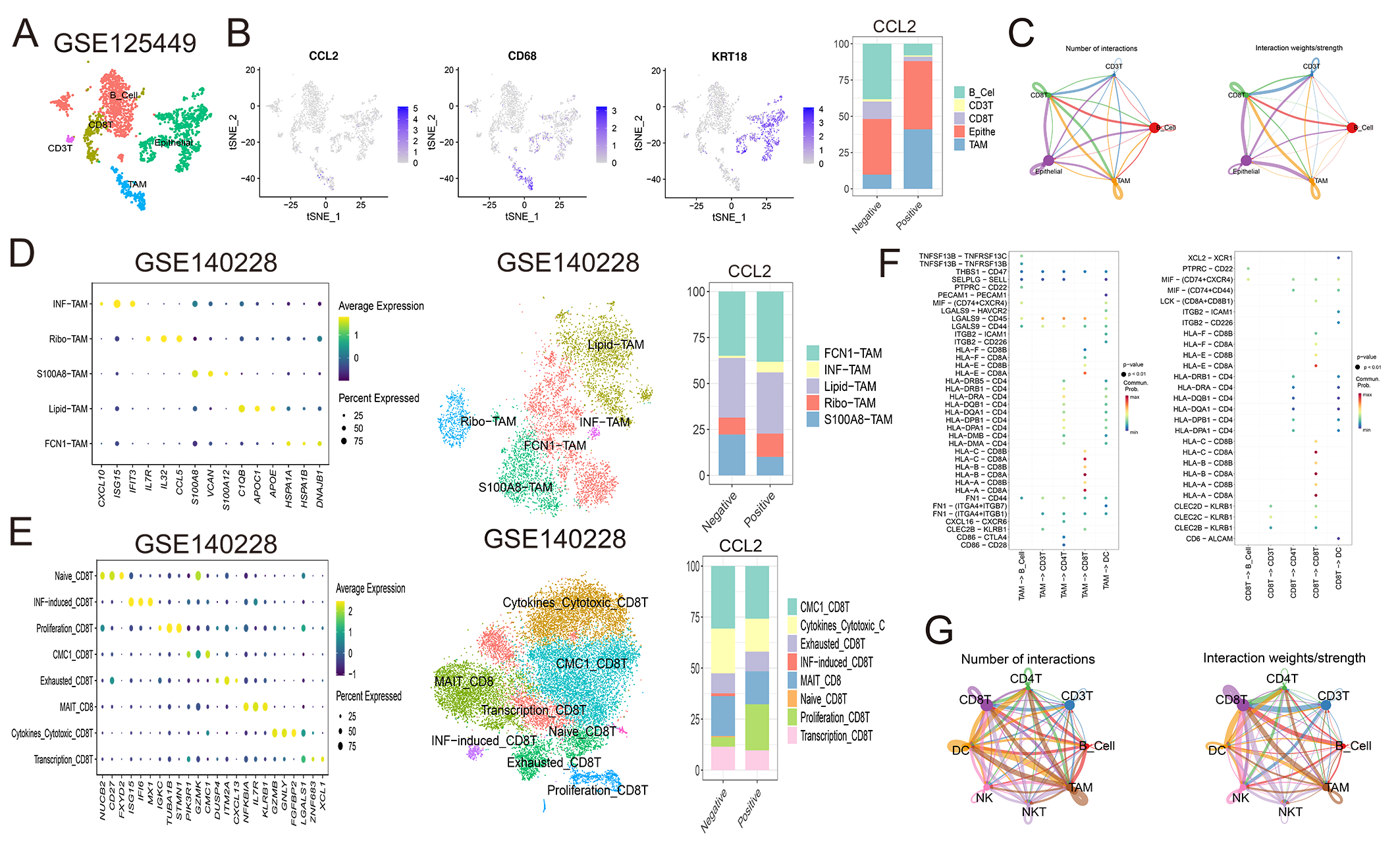

Supplement: Supplementary file 5 — Supplementary Material 5 [file 12943_2024_2049_MOESM5_ESM.tif]

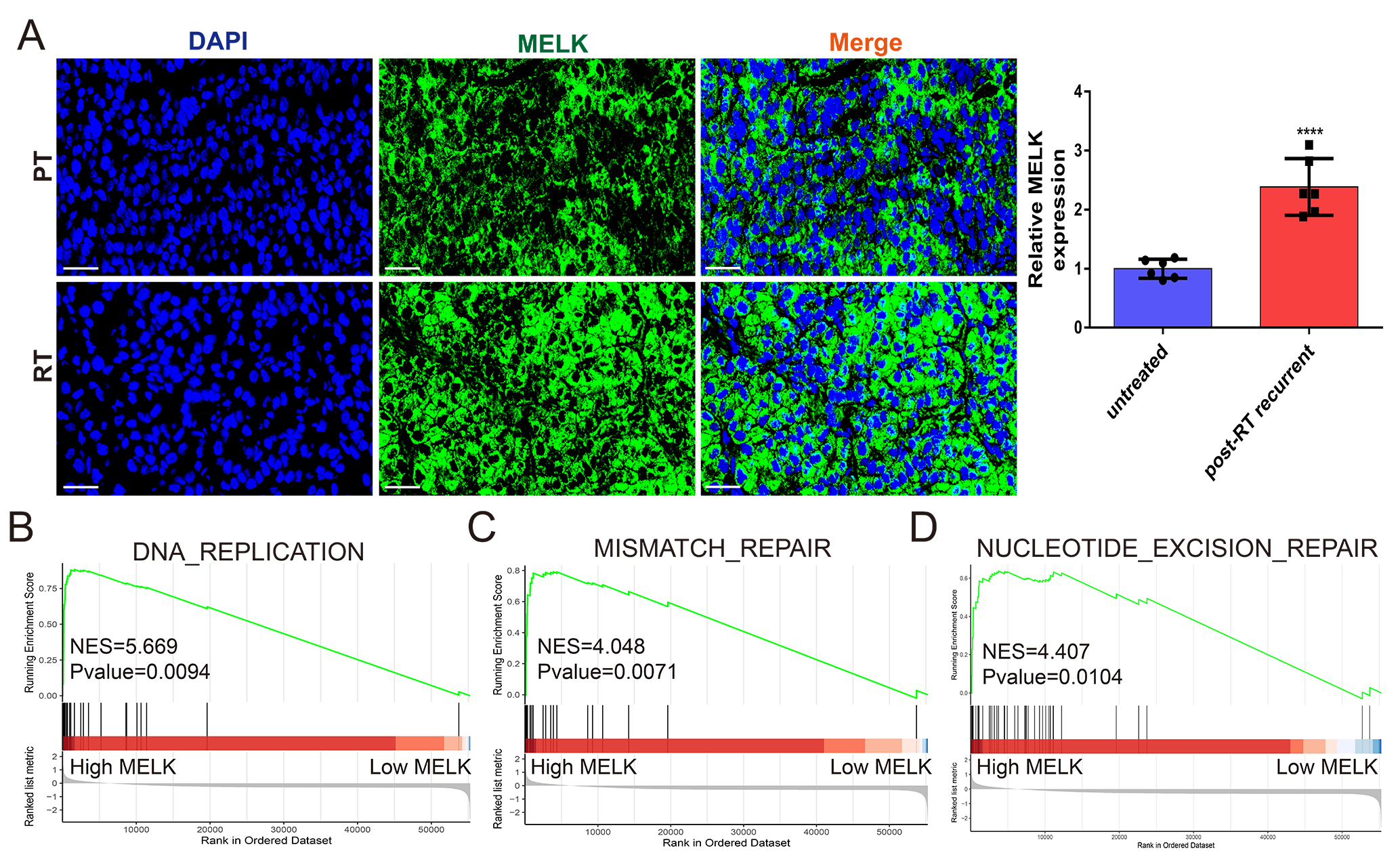

Supplement: Supplementary file 6 — Supplementary Material 6 [file 12943_2024_2049_MOESM6_ESM.tif]

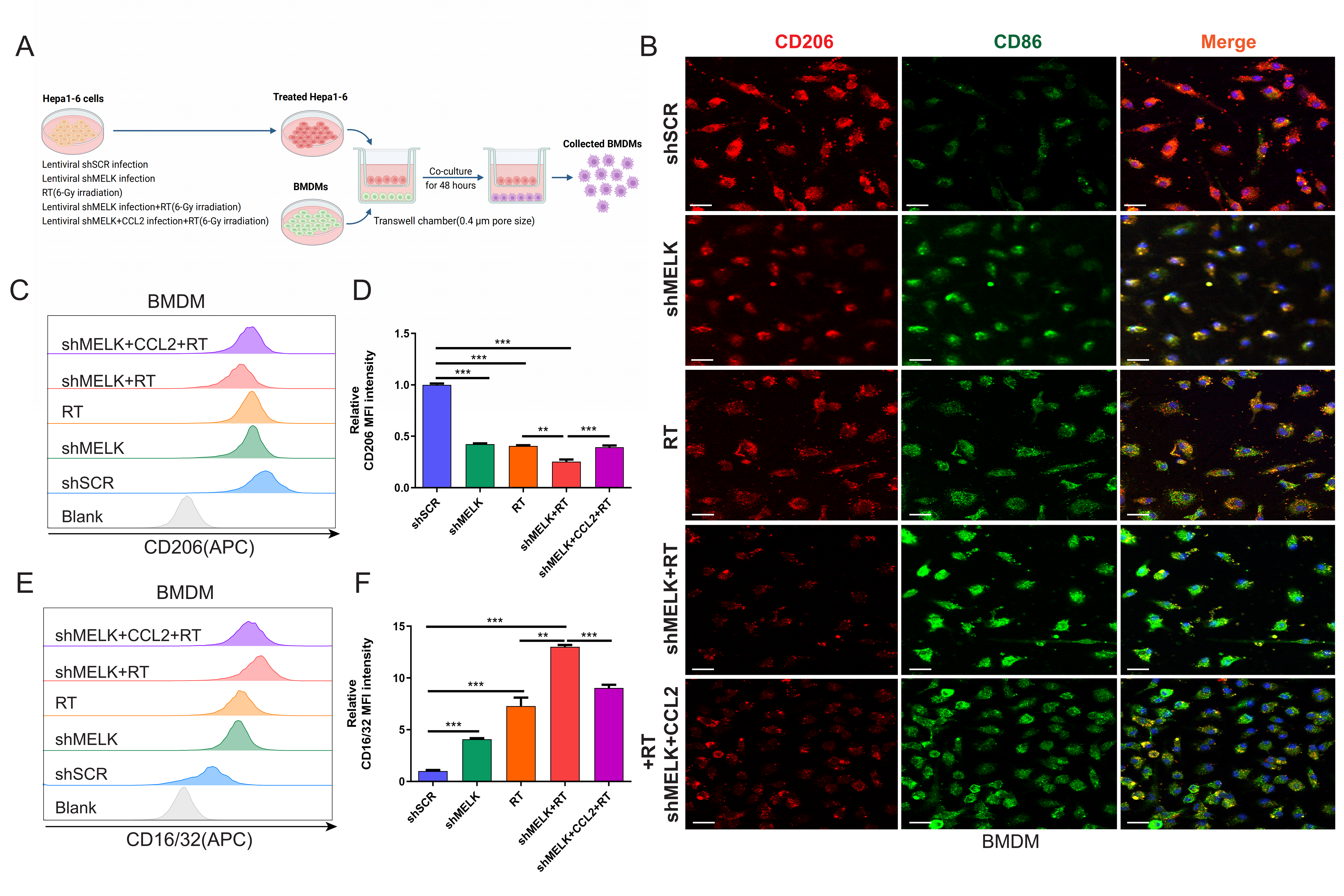

Supplement: Supplementary file 7 — Supplementary Material 7 [file 12943_2024_2049_MOESM7_ESM.tif]

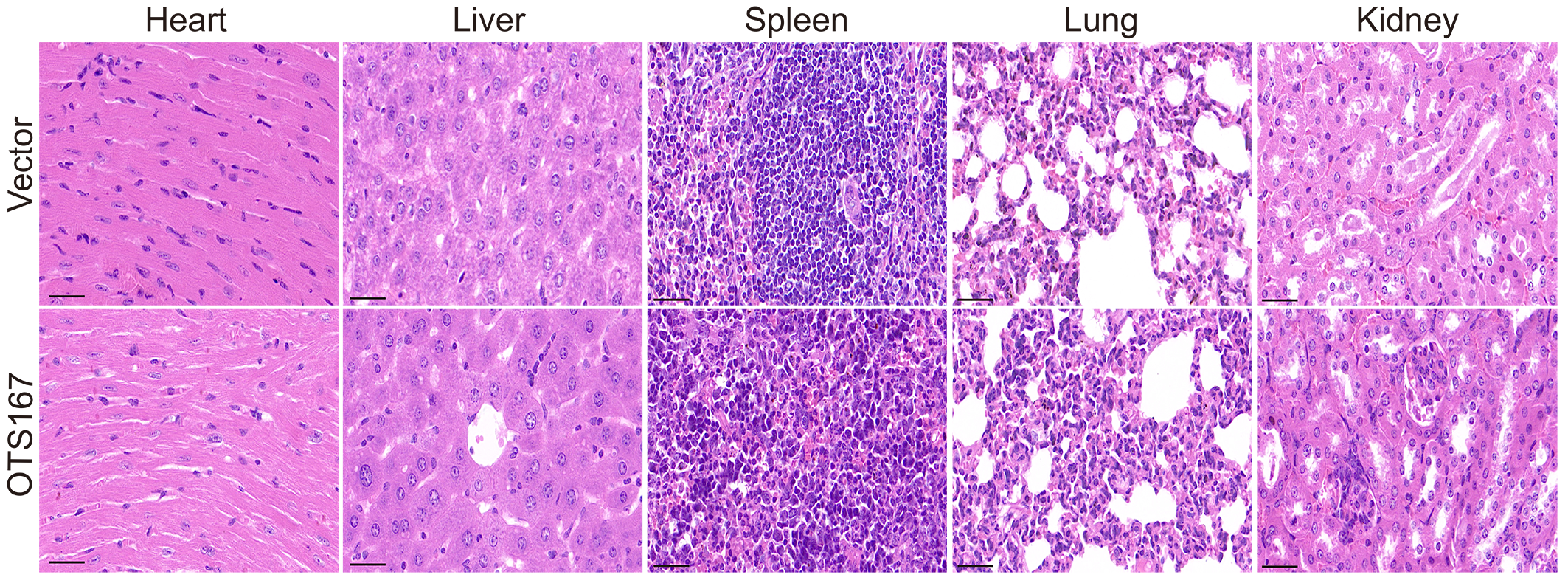

Supplement: Supplementary file 8 — Supplementary Material 8 [file 12943_2024_2049_MOESM8_ESM.tif]

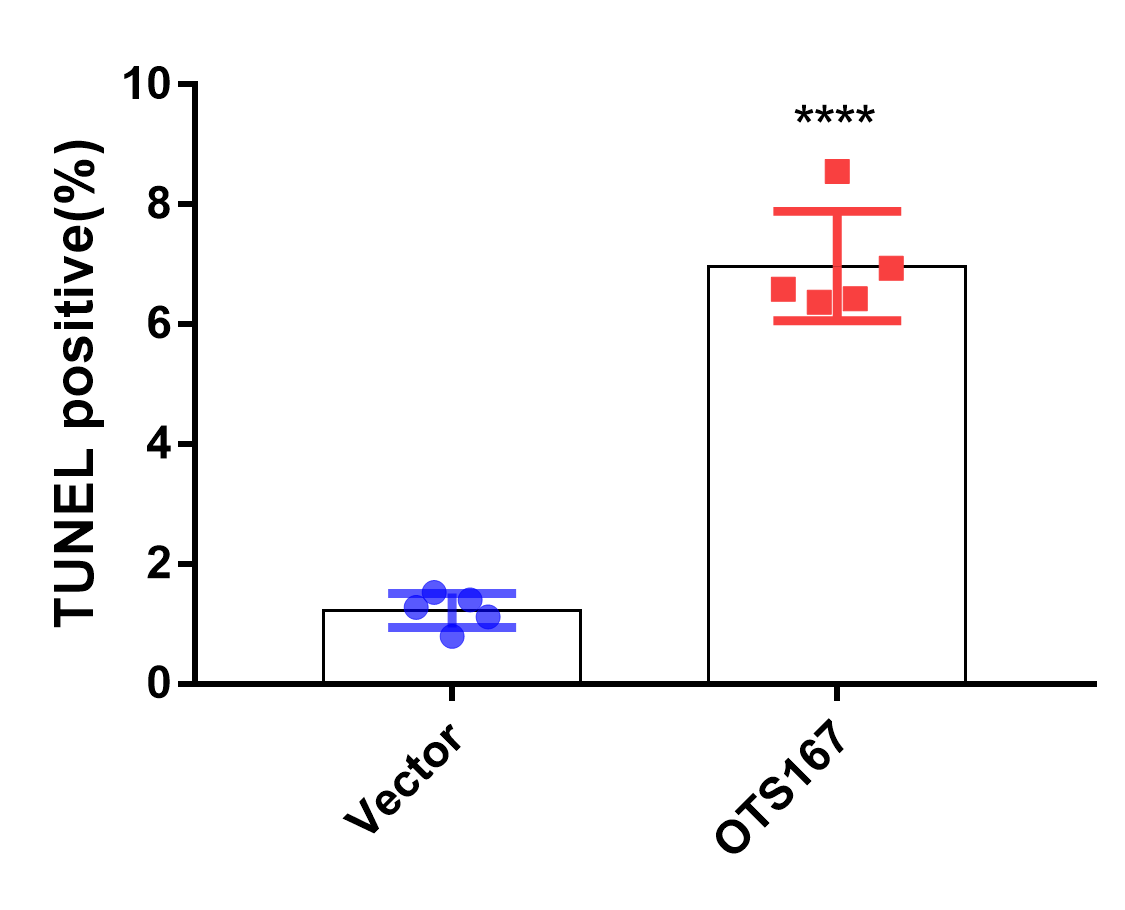

Supplement: Supplementary file 9 — Supplementary Material 9 [file 12943_2024_2049_MOESM9_ESM.tif]
