# Supplementary material for: Tumor cell-intrinsic MELK enhanced CCL2-dependent immunosuppression to exacerbate hepatocarcinogenesis and confer resistance of HCC to radiotherapy
Source: Mol Cancer. 2024 Jul 5;23:137. doi: 10.1186/s12943-024-02049-0 (PMC11225310; doi:10.1186/s12943-024-02049-0)
Supplement: Supplementary file 10 — Supplementary Material 10 [file 12943_2024_2049_MOESM10_ESM.doc]

**Supplementary materials**

**Figure S1. Elevated MELK expression in HCC effectively predicts poor disease outcomes in HCC patients. (A-B)** An overall view of the expression characteristics of MELK in tumors in different organs from the TCGA database. **(C)** Survival analysis showing that high MELK expression predicts worse DFS in HCC patients than low MELK expression. **(D-E)** ROC curves revealing the prognostic predictive performance of MELK expression in the TCGA-LIHC cohort (D) and ICGC cohort (E). **(F)** Identification of prognostic predictive factors of HCC in the TCGA-LIHC cohort. **(G)** The construction of a nomogram integrating the prognostic factors of HCC in the TCGA-LIHC cohort. **(H-J)** Calibration curves reflecting the consistency between the predicted prognosis and actual result.

**Figure S2. CRISPR/Cas9-mediated MELK knockout suppresses the proliferation of HCC cells. (A-B)** WB assay reflecting CRISPR/Cas9-mediated disruption of MELK in SK-HEP1 (A) and HCC-LM3 (B) cells. **(C)** The role of MELK knockout in colony formation in HCC cells.* p<0.05, ** p<0.01, *** p<0.001, **** p<0.0001.

**Figure S3. MELK inhibition hampers the tumorigenesis and apoptosis of HCC tissues. (A-D)** The effect of MELK knockdown on the expression of MELK (A), Ki67 (B), N-cadherin (C) and cleaved Casp3 (D) in HCC tissues. **(E)** The role of MELK deficiency in HCC apoptosis. *** p<0.001, **** p<0.0001.

**Figure S4. Effect of miR-505-3p on MELK expression.**

**Figure S5. The correlation between CCL2 expression and TME of HCC. (A)** The immune cellular landscape of HCC in the GSE125449 cohort. **(B)** The expression profiles of CCL2 in tumor cells and different immune cells in HCC. **(C)** Cellular communication relationships between different cells in GSE125449. **(D-E)** Expression profiles of CCL2 in different TAMs and CD8+T subgroups in GSE140228. **(F)** Cell communication between TAMs and other cells, and cell communication between CD8+ T cells and other cells in GSE140228. **(G)** Cellular communication relationships between different cells in GSE140228.

**Figure S6. The association between MELK expression and the clinical antitumor effect of RT. (A)** IF assay showing the expression changes of MELK in recurrent HCC tissues following RT. **(B-D)** GSEA analyses reflecting the close relationship between MELK and activation of signaling pathways, including “DNA replication” (B), “mismatch repair” (C) and “nucleotide excision repair” (D).

**Figure S7. MELK inhibition strengthens the anticancer effect of RT by regulating CCL2-mediated immune cell infiltration. (A)** Treatment illustration of the coculture system (drawn by https://biorender.com/). **(B)** IF assay reflecting the expression changes of CD206 and CD86 in BMDMs in response to different treatments. **(C-F)** FCM analysis showing the expression of CD16/32 (C-D) and CD206 (E-F) in Hepa1-6-cocultured BMDMs in response to different treatments. ** p<0.01, *** p<0.001.

**Figure S8. Pathological changes in heart, liver, spleen, lung and kidney upon OST167 treatment.**

**Figure S9. The effect of OTS167 treatment on HCC apoptosis.**
