# Supplementary material for: Tumor cell-intrinsic MELK enhanced CCL2-dependent immunosuppression to exacerbate hepatocarcinogenesis and confer resistance of HCC to radiotherapy
Source: Mol Cancer. 2024 Jul 5;23:137. doi: 10.1186/s12943-024-02049-0 (PMC11225310; doi:10.1186/s12943-024-02049-0)
Supplement: Supplementary file 11 — Supplementary Material 11 [file 12943_2024_2049_MOESM11_ESM.doc]

**1.1 Antibodies**

| Name | Dilution | Species | Manufacture | Cat, No. | Application |
| --- | --- | --- | --- | --- | --- |
| MELK | 1:1000 | Rabbit | proteintech | 11403-1-AP | Western blot |
|  | 1:1000 |  | Abcam | ab245574 | Western blot |
|  | / |  |  |  | IP |
|  | 1:400 |  |  | ab129373 | IF |
| STAT3 | 1:1000 | Rabbit | Abcam | ab32500 | Western blot |
|  | / |  |  |  | IP |
| Phosphor-STAT3（S727） | 1:1000 | Rabbit | Abcam | ab32143 | Western blot |
|  | 1:500 |  |  |  | IF |
| MCP1/CCL2 | 1:1000 | Rabbit | Abcam | ab214819 | Western blot |
|  | 1:50 |  |  |  | IF |
|  | 1:1000 | Rabbit | Proteintech | 26161-1-AP | Western blot |
| Vimentin | 1:1000 | Rabbit | [Cell Signaling Technology](https://www.baidu.com/link?url=ne_k8s24UxhaxCv9Y_LP58qZzV41MHKhKZy9Ul9MGxKZIu2VwbSZ79Ryp1xAd6jS&wd=&eqid=e96abed800009992000000046043551d) | #5741 | Western blot |
|  | 1:200 |  |  |  | IF |
| PCNA | 1:1000 | Rabbit | Cell Signaling  Technology | #13110 | Western blot |
|  | 1:400 |  |  |  | IF |
| MMP1 | 1:1000 | Rabbit | [Cell Signaling Technology](https://www.baidu.com/link?url=ne_k8s24UxhaxCv9Y_LP58qZzV41MHKhKZy9Ul9MGxKZIu2VwbSZ79Ryp1xAd6jS&wd=&eqid=e96abed800009992000000046043551d) | #54376 | Western blot |
| Ki67 | 1:400 | Rabbit | Cell Signaling Technology | #9027 | IHC |
| CD86 | 1:100 | Rabbit | Abcam | ab239075 | IF |
| N-cadherin | 1:1000 | Rabbit | Cell Signaling Technology | #13116 | Western blot |
|  | 1:200 |  |  |  | IHC |
| Cleaved Caspase-3 | 1:400 | Rabbit | Cell Signaling Technology | #9661 | IHC |
| IL-1β | 1:1000 | Rabbit | Proteintech | 16806-1-AP | Western blot |
| F4/80 | 1:400 | Rabbit | [Cell Signaling Technology](https://www.baidu.com/link?url=ne_k8s24UxhaxCv9Y_LP58qZzV41MHKhKZy9Ul9MGxKZIu2VwbSZ79Ryp1xAd6jS&wd=&eqid=e96abed800009992000000046043551d) | #30325 | IF |
| CD206 | 1:400 | Rabbit | [Cell Signaling Technology](https://www.baidu.com/link?url=ne_k8s24UxhaxCv9Y_LP58qZzV41MHKhKZy9Ul9MGxKZIu2VwbSZ79Ryp1xAd6jS&wd=&eqid=e96abed800009992000000046043551d) | #24595 | IF |
| Arginase-1 | 1:50 | Rabbit | [Cell Signaling Technology](https://www.baidu.com/link?url=ne_k8s24UxhaxCv9Y_LP58qZzV41MHKhKZy9Ul9MGxKZIu2VwbSZ79Ryp1xAd6jS&wd=&eqid=e96abed800009992000000046043551d) | #93668 | IF |
| β-Actin | 1:4000 | Mouse | Abcam | ab8226 | Western blot |
| Goat anti Rabbit IgG-HRP | 1:10000 | Goat | ABclonal Technology | AS014 | Western blot |
| Goat anti Mouse IgG-HRP | 1:10000 | Goat | ABclonal Technology | AS003 | Western blot |

**1.2 FACS antibodies**

| Name | Fluorophore | Clone | Manufacture | Cat, No. |
| --- | --- | --- | --- | --- |
| F4/80 | PE | BM8 | Biolegend | 123110 |
| CD206 | FITC | [C068C2](https://www.biolegend.com/en-gb/search-results?Clone=C068C2) | Biolegend | 141703 |
| CD45 | APC | [30-F11](https://www.biolegend.com/en-gb/search-results?Clone=30-F11) | Biolegend | 103112 |
| CD11b | FITC | M1/70 | Biolegend | 101205 |
| CD16/32 | FITC | 93 | Biolegend | 101306 |
| CD8 | FITC | 5H10-1 | Biolegend | 100803 |
| CD3 | APC | 17A2 | Biolegend | 100236 |
| CD4 | PE | [GK1.5](https://www.biolegend.com/en-gb/search-results?Clone=GK1.5) | Biolegend | 100408 |
| MHC class II | PE | HIS19 | Fitzgerald | 61R-1285 |

**1.3 qRT-PCR primers**

| Name |  | Sequence | Supplier |
| --- | --- | --- | --- |
| h-LIF | Forward | AATGAAGGTCTTGGCGGCAG | TSINGKE Biological Technology, Beijing, China. |
|  | Reverse | GTGACATGGGTGGCGTATGG |  |
| h-IL10 | Forward | TCTCTCACTCACCTTTGGCTC |  |
|  | Reverse | TGGGTCTTGGTTCTCAGCTT |  |
| h-IL1b | Forward | TTCGAGGCACAAGGCACAA |  |
|  | Reverse | CCATCATTTCACTGGCGAGC |  |
| h-IL6 | Forward | AGGGAGCGATAAACACAAACTCT |  |
|  | Reverse | CAGGGAGAAGGCAACTGGAC |  |
| h-CCL2 | Forward | CCTTCATTCCCCAAGGGCTC |  |
|  | Reverse | CTTCTTTGGGACACTTGCTGC |  |
| m-TNFα | Forward | GTAGCCCACGTCGTAGCAAA |  |
|  | Reverse | ACAAGGTACAACCCATCGGC |  |
| m-IL1β | Forward | TGCCACCTTTTGACAGTGATG |  |
|  | Reverse | ATGTGCTGCTGCGAGATTTG |  |
| m-IL12 | Forward | ACCCTTGCATCTGGCGTCTA |  |
|  | Reverse | TGGTTTAGGAGGGCAAGGGT |  |
| m-ARG1 | Forward | ACATTGGCTTGCGAGACGTA |  |
|  | Reverse | ATCACCTTGCCAATCCCCAG |  |
| m-MGL2 | Forward | ACTTCCAGAACTTGGAGCGG |  |
|  | Reverse | ACTGGGAAGGAATTAGAGCAAACT |  |
| m-MMP2 | Forward | CAAGTTCCCCGGCGATGTC |  |
|  | Reverse | TTCTGGTCAAGGTCACCTGTC |  |
| m-MRC1 | Forward | GGCTGATTACGAGCAGTGGA |  |
|  | Reverse | ACATGCCAGGGTCACCTTTC |  |
| m-YM1 | Forward | GCAGAAGCTCTCCAGAAGCAAT |  |
|  | Reverse | TCCCTTCTATTGGCCTGTCCT |  |
| m-TGF-β | Forward | CTCCCGTGGCTTCTAGTGC |  |
|  | Reverse | GCCTTAGTTTGGACAGGATCTG |  |
| m-CXCL10 | Forward | AAGCTATGTGGAGGTGCGAC |  |
|  | Reverse | AACCCCTTGGGAAGATGGTG |  |
| m-CXCL11 | Forward | CCACGCTACCTTCTGTGGTT |  |
|  | Reverse | ATGTTCGTGTGCCTCGTGAT |  |
| h-actin | Forward | CATGTACGTTGCTATCCAGGC |  |
|  | Reverse | CTCCTTAATGTCACGCACGAT |  |
| m-actin | Forward | TGAGCTGCGTTTTACACCCT |  |
|  | Reverse | GCCTTCACCGTTCCAGTTTT |  |

**1.4 Target sequences of sgRNA**

| Name |  | Sequence | Supplier |
| --- | --- | --- | --- |
| MELK | Sequence 1 | TGGGACAGGTGGCTTTGCAA | TSINGKE Biological Technology, Beijing, China |
|  | Sequence 2 | GAATGGAGGAGATGATCCGGTA |  |
