# Supplementary material for: Tumor cell-intrinsic MELK enhanced CCL2-dependent immunosuppression to exacerbate hepatocarcinogenesis and confer resistance of HCC to radiotherapy
Source: Mol Cancer. 2024 Jul 5;23:137. doi: 10.1186/s12943-024-02049-0 (PMC11225310; doi:10.1186/s12943-024-02049-0)
Supplement: Supplementary file 12 — Supplementary Material 12 [file 12943_2024_2049_MOESM12_ESM.doc]

**Table S1. Correlation between the expression of MELK and clinicalpathological characteristics in HCC patients from the validation cohort**

| Clinicopathological characterisitcs | MELK expression | | P-value |
| --- | --- | --- | --- |
|  | Low (N=34) | High(N=34) |  |
| **Age(years)** |  | | |
| <65 | 28 | 32 | 0.1362 |
| >=65 | 6 | 2 |  |
| **Gender:** |  | |  |
| Male | 31 | 30 | **0.0047** |
| Female | 3 | 4 |  |
| **HBV** |  | | |
| Negative | 2 | 1 | 0.5617 |
| Positive | 32 | 33 |  |
| **AFP** |  |  |  |
| <400 | 19 | 16 | **0.0013** |
| >=400 | 15 | 18 |  |
| **Grade** |  |  |  |
| G1 | 5 | 4 | 0.1337 |
| G2 | 21 | 17 |  |
| G3 | 8 | 13 |  |
| **TNM stage** |  | | |
| Ⅰ | 11 | 12 | **<0.0001** |
| Ⅱ | 4 | 3 |  |
| Ⅲ | 11 | 10 |  |
| Ⅳ | 8 | 9 |  |
| **Vascular invasion** |  | | |
| Yes | 9 | 10 | **0.0025** |
| No | 25 | 24 |  |
| **Recurrence** |  |  |  |
| Yes | 19 | 13 | 0.1493 |
| No | 15 | 21 |  |
